# What Makes a Mimic? Orange, Red, and Black Color Production in the Mimic Poison Frog (*Ranitomeya imitator*)

**DOI:** 10.1093/gbe/evae123

**Published:** 2024-06-28

**Authors:** Andrew O Rubio, Adam M M Stuckert, BreAnn Geralds, Rasmus Nielsen, Matthew D MacManes, Kyle Summers

**Affiliations:** Department of Biology, East Carolina University, Greenville, NC 27858, USA; Department of Biology and Biochemistry, University of Houston, Houston, TX 77204, USA; Department of Biology and Biochemistry, University of Houston, Houston, TX 77204, USA; Department of Biology, East Carolina University, Greenville, NC 27858, USA; School of Biological Sciences, Southern Illinois University, Carbondale, IL 62901, USA; Department of Integrative Biology, University of California, Berkeley, CA 94720, USA; Department of Molecular, Cellular, and Biomedical Sciences, University of New Hampshire, Durham, NH 03824, USA; Department of Biology, East Carolina University, Greenville, NC 27858, USA

**Keywords:** amphibian, aposematism, coloration genetics, gene expression, genomics

## Abstract

Aposematic organisms rely on their conspicuous appearance to signal that they are defended and unpalatable. Such phenotypes are strongly tied to survival and reproduction. Aposematic colors and patterns are highly variable; however, the genetic, biochemical, and physiological mechanisms producing this conspicuous coloration remain largely unidentified. Here, we identify genes potentially affecting color variation in two color morphs of *Ranitomeya imitator*: the orange-banded Sauce and the redheaded Varadero morphs. We examine gene expression in black and orange skin patches from the Sauce morph and black and red skin patches from the Varadero morph. We identified genes differentially expressed between skin patches, including those that are involved in melanin synthesis (e.g. *mlana, pmel, tyrp1*), iridophore development (e.g. *paics, ppat, ak1*), pteridine synthesis (e.g. *gch1, pax3-a, xdh*), and carotenoid metabolism (e.g. *dgat2, rbp1, scarb2*). In addition, using weighted correlation network analysis, we identified the top 50 genes with high connectivity from the most significant network associated with gene expression differences between color morphs. Of these 50 genes, 13 were known to be related to color production (*gch1, gmps, gpr143, impdh1, mc1r, pax3-a, pax7, ppat, rab27a, rlbp1, tfec, trpm1, xdh*).

**Significance**

The genetic basis producing aposematic coloration largely remains understudied. Here, we explore genes that are differentially expressed in orange, red, and black skin of the mimic poison frog, *Ranitomeya imitator*, which has undergone a Mullerian “mimetic radiation” across northern Peru. Our results revealed the differential expression of several genes previously associated with color pattern development. These findings allow us to identify key genes and pathways underlying Mullerian mimicry.

## Introduction

Across the tree of life, diverse color patterns have evolved as central elements of key ecological and evolutionary processes, ranging from species recognition (Losos 1985) to dynamic signaling in mate choice (Kokko et al. 2002), to predator avoidance (Ruxton et al. 2004). The selective advantages of certain phenotypes can lead to the evolution of mimicry, such as Batesian mimicry between coral snakes and scarlet kingsnakes (Savage and Slowinski 1992) or Mullerian mimicry among *Heliconius* butterflies (Mallet and Joron 1999). Because color phenotypes are often strongly correlated with survival and reproduction, the genetic, biochemical, and physiological mechanisms controlling color and pattern variation have long been of interest to evolutionary biologists.

Until recently, research focused on the genetic underpinning of color pattern diversity and molecular pathways in vertebrates have been aimed mainly at melanin-based coloration of mammals, birds, and fish (San-Jose and Roulin 2017; Elkin et al. 2023). The array of colors and patterns displayed by amphibians, reptiles, and fish are associated with chromatophores (organelles), including melanophores, iridophores, and xanthophores (Bagnara 1966). Chromatophores can contain pigments (such as melanins or pteridines) and structural elements (such as guanine platelets). Dark coloration, such as black, brown, and dark green, is produced by melanophores and melanin pigments (Duellman and Trueb 1994). Structural coloration is largely determined by the size, shape, and orientation of reflective platelets in iridophores. For example, thin platelets reflect wavelengths in the dark blue part of the visual spectrum, and thicker platelets (in conjunction with pigmentation) can produce orange coloration (2020). Production of yellow, red, and orange colors is associated with pteridine and carotenoid pigments contained in xanthophores (Obika and Bagnara 1964; Grether et al. 2001; Mcgraw et al. 2006; Croucher et al. 2013; McLean et al. 2017). The genetic basis of carotenoid- and pteridine-based color production has been a focus of investigation recently, using new genomic methods (e.g. Hooper et al. 2019; Ahi et al. 2020). Twomey et al. (2020b) found that differences in brightness and hue are the results of interactions between pigments, such as carotenoids and pteridines, and structural elements, such as guanine platelets.

Aposematic organisms use visual cues to communicate that they are chemically defended and unpalatable (Harvey et al. 1982; Brown 2013; Cummings and Crothers 2013), and identifying the genetic basis of coloration in aposematic species has been a major emphasis of recent research (e.g. Liu et al. 2014; Posso-Terranova and Andrés 2017; Stuckert et al. 2019; Rodríguez et al. 2020; Stuckert et al. 2024; Twomey, et al. 2020a). In addition, several studies have identified genes related to color development under positive selection in aposematic species (Rodríguez et al. 2020; Linderoth et al. 2023; Rubio et al. 2024). Historically, evolutionary biologists have predicted that aposematism, driven by predator-imposed selective pressures, causes sympatric phenotypes among closely related species to converge on a single color pattern to promote accelerated predator learning (Müller 1879; Mallet and Joron 1999). However, geographic variation in both color and pattern is common in aposematic species (Joron and Mallet 1998; Briolat et al. 2019, 2018), potentially driven by mate choice, intraspecific aggression, or differential predator selective regimes (Summers et al. 1997; Jiggins et al. 2004; Lawrence et al. 2019; Bieri et al. 2024).

Approximately one-third of Neotropical poison frogs (Amphibia: Dendrobatidae) are thought to be aposematic (Summers and Clough 2001; Santos et al. 2003). This group of frogs are interesting models for the examination of color production because colors of the aposematic phenotypes are clearly tied to selection and because there is extensive variation of color phenotypes within the family and within species. The polytypic mimic poison frog, *Ranitomeya imitator*, provides an important example of variation in color pattern. This species has undergone a Mullerian “mimetic radiation” across different geographic regions of Northern Peru (Symula et al. 2001, 2003). To date, there are four known *R. imitator* color morphs that occur in sympatry with three congeneric model species (Symula et al. 2001; Stuckert et al. 2014). Significant progress has been made in identifying the genes underlying color production in poison frogs (Stuckert et al. 2019; Twomey, et al. 2020a; Rodríguez et al. 2020; Linderoth et al. 2023). Of particular relevance is a recent study by Stuckert et al. (2024) which examined gene expression of whole-skin samples from *R. imitator*, *Ranitomeya fantastica*, and *Ranitomeya variabilis.* This study identified a number of crucial genes and pathways likely playing a role in producing these different color patterns, but at a broad, whole skin-wide scale. Thus, further work, specific to distinct, differently colored patches of skin, is required to identify key genes and pathways underlying Mullerian mimicry in these frogs.

**Fig. 1.**
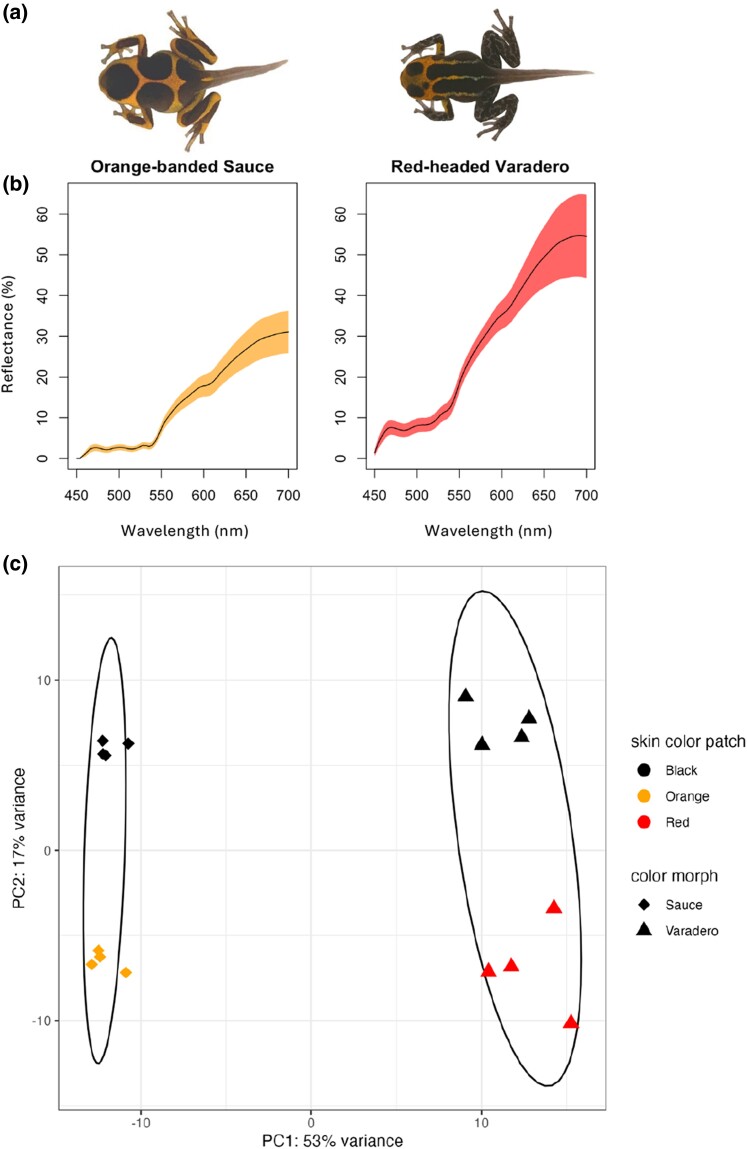
Overview of study system. a) Depictions of captive *R. imitator* froglet morphs used in this study. From left to right: the Sauce morph and the Varadero morph. b) Mean reflectance spectra including SD of bright dorsal skin patches (Sauce morph: orange and Varadero morph: red) from parents of the froglets used in this study. Reflectance data were taken from the brightly colored areas in the dorsum of four adult frogs for each morph. c) Plot of the principal component analysis summarizing the expression patterns across samples. The ellipses are derived by a multivariate *t*-distribution and group skin patches by morph.

In this study, we use genomic tools to shed light on the genetic and biochemical mechanisms underlying skin color differences within and between two morphs of *R. imitator*. This is of general interest to evolutionary biologists, because *R. imitator* is a model system for the study of Mullerian mimicry (Mallet 2014). We investigated patterns of differential gene expression between three different dorsal skin patches (black, orange, and red) from two captive bred color morphs of the mimic poison frog (*R. imitator*), the orange-banded Sauce and redheaded Varadero morphs (Fig. 1a). The Sauce morph is characterized by orange bands on a black dorsum, while the Varadero morph is characterized by a red head and dorsolateral lines over a black dorsum. We used RNA-sequencing to quantify gene expression exclusively in the black and orange dorsal skin patches in the Sauce morph and black and red dorsal skin patches in the Varadero morph, to identify candidate genes involved with color production. We predicted that black dorsal skin patches in both the Sauce and Varadero color morph would have an upregulation of genes related to melanin synthesis. In contrast, we predicted that genes related to iridophore development, carotenoid metabolism, and pteridine synthesis would be upregulated in both the orange and red dorsal skin patches in the Sauce and Varadero morphs.

## Results

### Overall Gene Expression Differences

An average of 22.66 million (± 1.72 SD) sequence paired-end reads were generated per sample, and of these, an average of 84.12% (± 0.75% SD) of the reads mapped to the *R. imitator* genome. All gene expression count data are available on Zenodo (https://zenodo.org/records/11521755). A total of 22,963 genes were found to be expressed, 17,549 of which had a total experiment-wide expression level of ≥ 50. We found strong, distinct differences in gene expression between patches in both the Sauce and Varadero morphs and across morphs. In our principal component analysis (PCA), the first principal component, which explained 53% of the variation, was largely driven by color morph, whereas the second principal component, which explained 17% of the variation, was largely driven by skin color patch (Fig. 1c). When conducting a PCA within morphs, 47% of the variation was largely driven by black and orange skin patches in the Sauce morph (supplementary fig. S1, Supplementary Material online) and 35% of the variation was largely driven by black and red skin patches in the Varadero morph (supplementary fig. S2, Supplementary Material online).

### Sauce Morph: Black and Orange Skin Patches

**Fig. 2.**
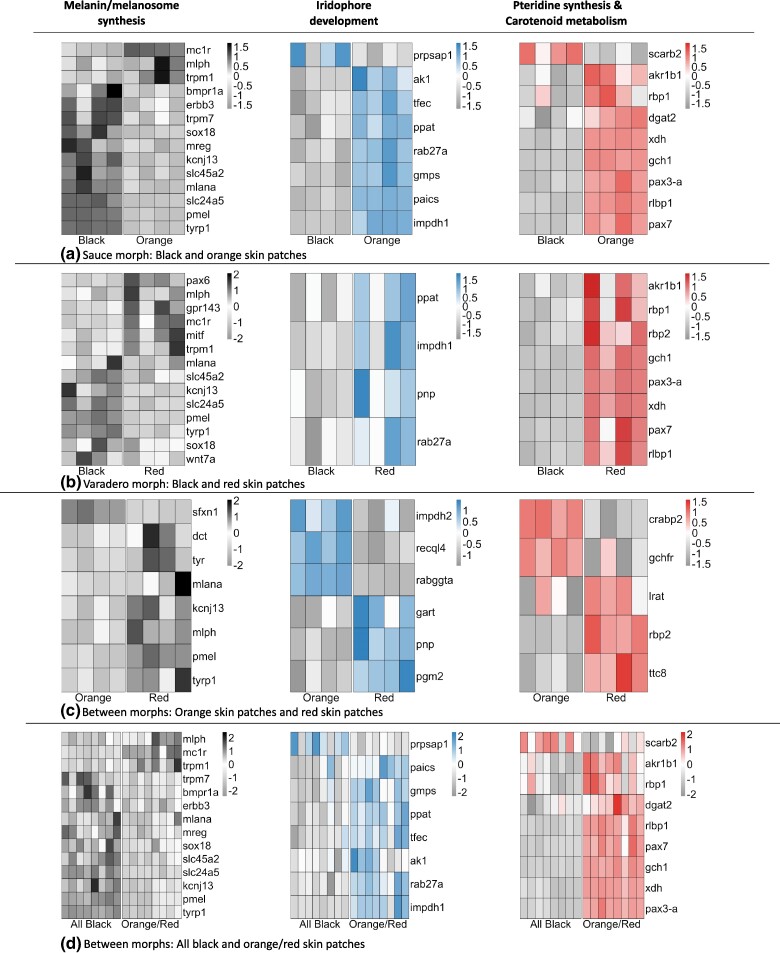
Heatmap generated by pheatmap summarizing differentially expressed (DE) color-related genes among a) black skin patches and orange skin patches of the Sauce morph, b) black skin patches and red skin patches of the Varadero morph, c) orange skin patches of the Sauce morph and red skin patches of the Varadero morph, and d) black skin patches and orange/red skin patches of both the Sauce and Varadero morph. DE genes related to melanocyte production and melanin synthesis are indicated with black coloration, DE genes related to iridophore development are indicated with blue coloration, and DE genes related to both pteridine synthesis and carotenoid metabolism are indicated with red coloration.

We identified 278 differentially expressed genes between the black and orange skin patches of frogs from the Sauce morph (supplementary table S1, Supplementary Material online). Of these differentially expressed genes, 46, from an a priori color gene list, have been putatively linked to pigment production in poison frogs or in other taxa (16.54% of genes that were differentially expressed were related to color production) (supplementary table S2, Supplementary Material online). Thirteen genes were upregulated in the black skin patches (Fig. 2a). Of these, 11 genes were related to melanocyte production and melanin synthesis (*bmpr1a, erbb3, kcnj13, mlana, mreg, pmel, slc24a5, slc45a2, sox18, tyrp1, trpm7*), one gene was related to iridophore development (*prpsap1*), and one gene was related to carotenoid metabolism (*scarb2*). Conversely, 18 genes were upregulated in the orange skin patches (Fig. 2a). Of these, three genes were related to melanocyte production and melanin synthesis (*mc1r, mlph, trpm1*), seven genes were related to iridophore development (*ak1, gmps, impdh1, paics, ppat, rab27a, tfec*), four genes were related to pteridine synthesis (*gch1, pax3-a, pax7, xdh*), and four genes were related to carotenoid metabolism (*akr1b1, dgat2, rbp1, rlbp1*).

### Varadero Morph: Black and red Skin Patches

We identified 350 differentially expressed genes between black and red skin patches of frogs from the Varadero morph (supplementary table S1, Supplementary Material online). Of these differentially expressed genes, 56, from an a priori color gene list, have been putatively linked to pigment production in poison frogs or in other taxa (16% of genes that were differentially expressed were related to color production) (supplementary table S2, Supplementary Material online). Eight genes were upregulated in black skin patches (Fig. 2b). All of these genes were related to melanocyte production and melanin synthesis (*kcnj13, mlana, pmel, slc24a5, slc45a2, sox18, tyrp1, wnt7a*). Conversely, 18 genes were upregulated in the red skin patches (Fig. 2b). Of these, six genes were related to melanocyte production and melanin synthesis (*gpr143*, *mc1r, mitf, mlph, pax6, trpm1*), four genes were related to iridophore development (*impdh1*, *pnp, ppat, rab27a*), four genes were related to pteridine synthesis (*gch1, pax3-a, pax7, xdh*), and four genes were related to carotenoid metabolism (*akr1b1, rbp1, rbp2, rlbp1*).

### Differentially Expressed Genes Between Skin Patches Shared Across Morphs

We identified 1,499 differentially expressed genes between orange skin patches of the Sauce morph and the red skin patches of the Varadero morph (supplementary table S1, Supplementary Material online). Of these differentially expressed genes, 116, from an a priori color gene list, have been putatively linked to pigment production in poison frogs or in other taxa (7.73% of genes that were differentially expressed were related to color production) (supplementary table S2, Supplementary Material online). Six genes were upregulated in orange skin patches (Fig. 2c). Of these, one gene was related to melanocyte production and melanin synthesis (*sfxn1*), three genes were related to iridophore development (*impdh2, rabggta, recql4*), one gene was related to pteridine synthesis (*gchfr*), and one gene was related to carotenoid metabolism (*crabp2*). Conversely, 12 genes were upregulated in the red skin patches (Fig. 2c). Of these, seven genes were related to melanocyte production and melanin synthesis (*dct, kcnj13, mlana, mlph, pmel, tyr, tyrp1*), three genes were related to iridophore development (*gart, pnp, pgm2*), and three genes were related to carotenoid metabolism (*lrat, rbp2, ttc8*). In addition, we identified 1,353 differentially expressed genes between black skin patches of the Sauce morph and black skin patches of the Varadero morph (supplementary table S1, Supplementary Material online). Of these differentially expressed genes, 97, from an a priori color gene list, have been putatively linked to pigment production in poison frogs or in other taxa (7.16% of genes that were differentially expressed were related to color production) (supplementary table S2, Supplementary Material online).

We identified 278 differentially expressed genes between black skin patches and orange/red skin patches from the Sauce and Varadero morph (supplementary table S1, Supplementary Material online). Of these differentially expressed genes, 46, from an a priori color gene list, have been putatively linked to pigment production in poison frogs or in other taxa (16.54% of genes that were differentially expressed were related to color production) (supplementary table S2, Supplementary Material online). Thirteen genes were upregulated in the black skin patches (Fig. 2d). Of these, 11 genes were related to melanocyte production and melanin synthesis (*bmpr1a, erbb3, kcnj13, mlana, mreg, pmel, slc24a5, slc45a2, sox18, tyrp1, trpm7*), one gene was related to iridophore development (*prpsap1*), and one gene was related to carotenoid metabolism (*scarb2*). Conversely, 18 genes were upregulated in the orange/red skin patches (Fig. 2d). Of these, three genes were related to melanocyte production and melanin synthesis (*mc1r, mlph, trpm1*), seven genes were related to iridophore development (*ak1, gmps, impdh1, paics, ppat, rab27a, tfec*), four genes were related to pteridine synthesis (*gch1, pax3-a, pax7, xdh*), and four genes were related to carotenoid metabolism (*akr1b1, dgat2, rbp1, rlbp1*).

We found 20 genes that showed similar patterns of differential expression in the comparison of black and orange/red skin patches in both the Sauce and Varadero morphs (Table 1: derived by cross matching differentially expressed genes from both within-morph comparisons; Fig. 2a, b). Of these genes, ten were related to melanocyte production and melanin synthesis (*mc1r, kcnj13, mlana, mlph, pmel, slc24a5, slc45a2, sox18, trpm1, tyrp1*), three were related to iridophore development (*impdh1, ppat, rab27a*), four were related to pteridine synthesis (*gch1, pax3-a, pax7, xdh*), and three were related to carotenoid metabolism (*akr1b1, rbp1, rlbp1*). Seven genes related to melanocyte production and melanin synthesis (*kcnj13, mlana, pmel, slc24a5, slc45a2, sox18, tyrp1*) were upregulated in the black skin patches of both the Sauce and Varadero morphs. All genes related to iridophore development (*impdh1*, *ppat, rab27a*), pteridine synthesis (*gch1, pax3-a, pax7, xdh*), and carotenoid metabolism (*akr1b1, rbp1, rlbp1*), in addition to three genes related to melanocyte and melanin synthesis (*mc1r, mlph, trpm1*), were upregulated in orange and red skin patches of both of these morphs.

**Table 1:** Differentially expressed genes that were shared between skin section comparisons from the Sauce and Varadero morphs

Gene	Sauce: Upregulated in skin patches	Varadero: Upregulated in skin patches	Coloration role	Citation
*mc1r*	Orange	Red	Melanin/melanosome synthesis	Selz et al. (2007)
*kcnj13*	Black	Black	Melanin/melanosome synthesis	Iwashita et al. (2006)
*mlana*	Black	Black	Melanin/melanosome synthesis	De Mazière et al. (2002)
*mlph*	Orange	Red	Melanin/melanosome synthesis	Cirera et al. (2013)
*pmel*	Black	Black	Melanin/melanosome synthesis	Schonthaler et al. (2005)
*slc24a5*	Black	Black	Melanin/melanosome synthesis	Lamason et al. (2005)
*slc45a2*	Black	Black	Melanin/melanosome synthesis	Fernandez et al. (2008)
*sox18*	Black	Black	Melanin/melanosome synthesis	Pennisi et al. (2000)
*trpm1*	Orange	Red	Melanin/melanosome synthesis	Oancea et al. (2009)
*tyrp1*	Black	Black	Melanin/melanosome synthesis	Murisier and Beermann (2006)
*impdh1*	Orange	Red	Iridophore/guanine platelet	Szekeres et al. (1992)
*ppat*	Orange	Red	Iridophore/guanine platelet	An et al. (2008)
*rab27a*	Orange	Red	Iridophore/guanine platelet	Koike et al. (2019)
*gch1*	Orange	Red	Pteridine gene	Ziegler et al. (2000)
*pax3-a*	Orange	Red	Pteridine gene	Minchin and Hughes (2008)
*pax7*	Orange	Red	Pteridine gene	Nord et al. (2016)
*xdh*	Orange	Red	Pteridine gene	Reaume et al. (1991)
*akr1b1*	Orange	Red	Carotenoid metabolism	Ruiz et al. (2012)
*rbp1*	Orange	Red	Carotenoid metabolism	Fierce et al. (2008)
*rlbp1*	Orange	Red	Carotenoid metabolism	Saari and Crabb (2005)

Coloration role column indicates the color production.

### Weighted Correlation Network Analyses

Our weighted correlation network analysis (WGCNA) identified 19 gene modules in our expression data. Of these networks, nine modules were significantly correlated with differences between black and orange/red skin patches: module 2 (*r* = 0.66, *P*-value = 0.005), module 7 (*r* = 0.82, *P*-value< 0.000), module 9 (*r* = 0.92, *P*-value < 0.000), module 12 (*r* = −0.75, *P*-value < 0.000), module 13 (*r* = −0.7, *P*-value = 0.003), module 15 (*r* = −0.59, *P*-value = 0.02), module 16 (*r* = −0.57, *P*-value = 0.02), module 17 (*r* = −0.65, *P*-value = 0.006), and module 18 (*r* = −0.98, *P*-value < 0.000) (supplementary fig. S3, Supplementary Material online). In addition, seven modules were significantly correlated with differences between color morphs: module 3 (*r* = −0.85, *P*-value < 0.000), module 4 (*r* = −0.68, *P*-value = 0.004), module 5 (*r* = −0.72, *P*-value = 0.002), module 6 (*r* = −0.67, *P*-value = 0.005), module 10 (*r* = 0.54, *P*-value = 0.03), module 14 (*r* = 0.96, *P*-value < 0.000), and module 15 (*r* = 0.65, *P*-value = 0.006) (supplementary fig. S3, Supplementary Material online).

**Fig. 3.**
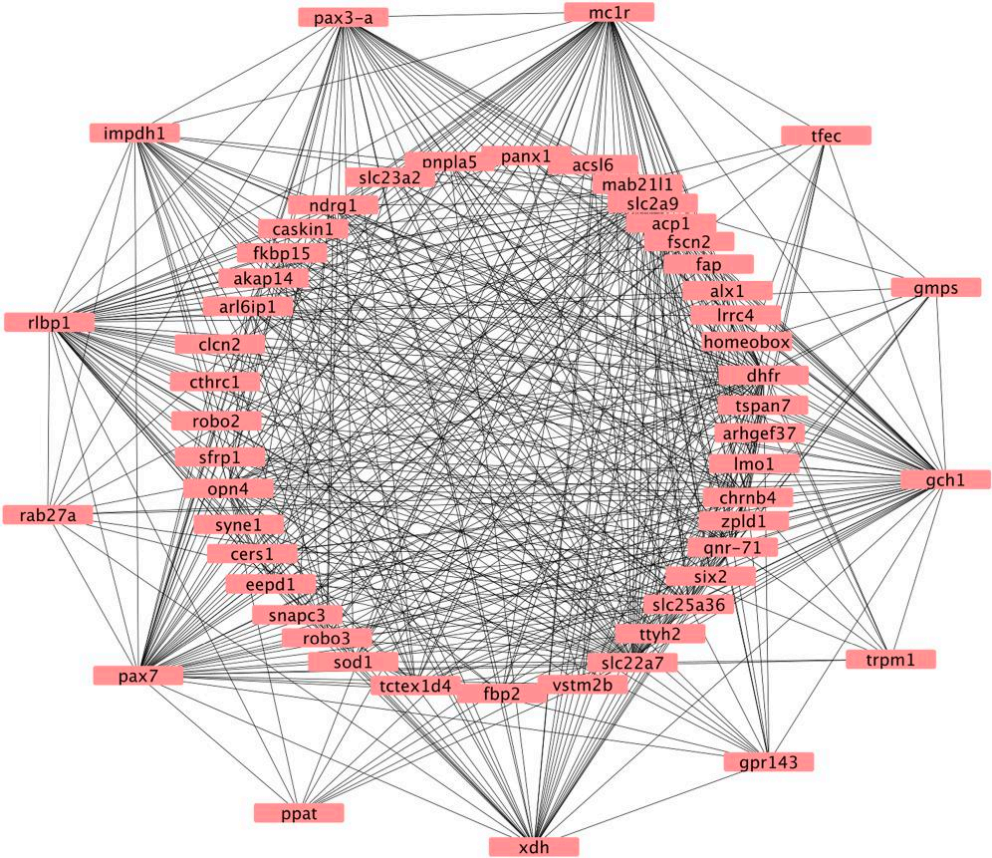
Hub genes from the most significant WGCNA module, module 14, correlated with color morph differences, Sauce and Varadero skin patches. The outer ring shows differentially expressed genes known to be involved in the development of color pattern from previous research.

We identified the top 50 genes with the highest connectivity in the module most correlated with color differences between black and orange/red skin patches, module 18 (*r* = −0.98). Of these, two genes were related to color production (*ikbkb, rabggta*). In addition, we identified the top 50 genes with the highest connectivity in the most significant module, module 14, correlated with color morph differences, Sauce and Varadero skin patches. Of these, 13 were related to color production (*gch1, gmps, gpr143, impdh1, mc1r, pax3-a, pax7, ppat, rab27a, rlbp1, tfec, trpm1, xdh*) (Fig. 3).

## Discussion

Research on the mechanisms controlling color pattern variation has accelerated in recent years (Orteu and Jiggins 2020). In this paper, we examined gene expression of individual color skin patches within the mimic poison frog *R. imitator* in an effort to identify genes that control differential color production. Our skin patch comparisons included two contrasts between black and orange/red skin patches within individuals (for individuals of the Sauce and Varadero morphs), as well as comparisons of the combined black skin patches and the combined orange/red skin patches (from both morphs), comparisons between the black skin patches of the Sauce and the Varadero morph, and comparisons between the orange and red skin patches of the Sauce and the Varadero morphs. This last comparison should reveal patterns of gene expression that are involved in subtle differences in hue (e.g. orange vs. red coloration), whereas comparisons between black and orange/red skin patches may highlight genes with more extreme effects on coloration (note that the expression patterns of some genes are likely to be involved in both types of effects).

Although there were large numbers of differentially expressed genes between the orange and red skin patches and between the black and black skin patches of the Sauce and Varadero morph, the proportion of color-related genes in these two comparison were smaller than the proportion of apriori color-related genes in the other three comparisons (Sauce black vs. Sauce orange: 16.54% of differentially expressed genes were related to color production; Varadero black vs. Varadero red: 16% of differentially expressed genes were related to color production; and all black vs. combined orange/red [from both morphs]: 16.54% of differentially expressed genes were related to color production, Sauce orange versus Sauce red: 7.73% of differentially expressed genes were related to color production, and Sauce black versus Varadero black: 7.16% of differentially expressed genes were related to color production). The large number of differentially expressed genes in these two comparisons is likely to be attributed to overall population differences. This work builds on that of Stuckert et al. (2024) which examined gene expression differences between morphs and across developmental stages in *R. imitator* and two model species (*R. variabilis* and *R. fantastica*). These researchers identified certain genes in several key pathways, including melanogenesis, guanine synthesis, and pteridine synthesis and hypothesized that keratin genes are also likely to be playing an important role in coloration in these species. Below we discuss specific color-related genes showing differential expression in our analyses. One of the most difficult tasks in analyzing differential expression data is organizing and interpreting the results. To do this, we first employed a curated list of color pattern genes gathered from the literature (McLean et al. 2017; Baxter et al. 2019) to identify genes known to be involved in color pattern development in other species. Second, we implemented a WGCNA which allowed us to identify the top 50 hub genes with the highest connectivity in the module most correlated with color morph differences, Sauce and Varadero skin patches. We organize our discussion of the results on the basis of known pathways involved in color development in specific layers of ectothermic vertebrate skin.

### Melanin/Melanosome Synthesis Pathways

Melanin-based coloration is ubiquitous among vertebrates and many genes affecting melanogenesis have been identified (Orteu and Jiggins 2020). Stuckert et al. (2024) found that many genes that form components of the melanogenesis pathway (including proliferation, migration and homing of melanoblasts, differentiation of melanocytes, melanosome formation, synthesis of eumelanin and putatively pheomelanin, and melanosome transport) were differentially expressed between color morphs and/or developmental stages. We note that many of these melanogenesis-related genes showed variation in expression associated with color variation in the green-and-black poison frog, *Dendrobates auratus* (Stuckert et al. 2019). Further, a number of these genes were also differentially expressed between color morphs or differently colored skin patches of the strawberry poison frog, *Oophaga pumilio* (Rodríguez et al. 2020; Stuckert et al. 2023). Fewer of these melanogenesis-related genes were differentially expressed between morphs in *O. pumilio*, as one might expect given that color morphs studied in that particular project lack black or gray coloration (Rodríguez et al. 2020), although many melanin-related genes were differentially expressed between dark green and yellow patches (Stuckert et al. 2023).

We identified several genes involved in the production of melanophores and melanin to be differentially expressed between skin patches (e.g. *erbb3, mitf, mlph, mc1r, mlana, pmel, pax6, slc24a5, trpm1, trpm7, tyrp1*) (Fig. 2). Abundant evidence from previous research has identified the important roles that these genes play in processes such as pigment cell migration from the neural crest, homing of melanoblasts, differentiation of melanocytes, melanosome formation, and melanogenesis (synthesis of melanin) itself. Many of these genes were upregulated in black skin patches relative to orange and/or red skin patches in both morphs (*kcnj13, mlana, pmel, slc24a5, slc45a2, sox18, tyrp1*) (Fig. 2 a, b, d). The genes *mlana, pmel,* and *tyrp1* were upregulated in red skin patches of the Varadero morph when compared with the orange skin patches of the Sauce morph (Fig. 2c). The *slc24a5* gene has long been known to affect pigmentation and is one of the major loci affecting skin color across human populations and in other vertebrates (Lamason et al. 2005). The tyrosinase-related protein 1 (*tyrp1*) is a key component of the melanogenesis pathway, interacting with tyrosine and various derivatives to produce melanin (Murisier and Beermann 2006). Premelanosome protein (*pmel*) is a well-known part of the melanosome synthesis pathway as it is crucial for the development of premelanosomes and also affects melanosome shape (Schonthaler et al. 2005). Mutation experiments with zebrafish *pmel* have produced hypopigmented individuals due to changes in the shape and melanin content of melanophores. This gene was also found to be a key locus influencing the development of striped patterning in cichlid fish (Ahi and Sefc 2017).

The genes *mitf* and *mlph* were found to be upregulated in orange and/or red skin patches relative to black skin patches (Fig. 2 a, b, d). The *mitf* locus has been described as the “master regulator” of melanogenesis and plays a central role in coordinating the production of melanocytes by regulating a variety of other genes (Kawakami and Fisher 2017). Given the role of *mitf* as a master regulator of multiple pathways of melanogenesis, its expression levels must be fine-tuned to balance multiple effects, and the significance of higher expression of this gene in orange/red skin patches is currently unclear. Computational gene network analyses of human pigment genes (Rambow et al. 2015) have revealed that *pmel, tyrp1,* and *mlana* are strongly regulated by *mitf* (melanocyte-inducing transcription factor).

A couple of genes that were downregulated in black skin patches relative to orange and/or red skin patches were identified as hub genes in our WGCNA/Cytoscape analyses (*mc1r, gpr143*) (Fig. 2a-b, d; Fig. 3). The G protein receptor-coupled protein 143 (*gpr143*) is not directly involved in melanogenesis, but nevertheless strongly influences melanic pigmentation, at least in mammals. This gene is central to various pigmentation diseases in the human retina (McKay 2019), and recent research demonstrates that it is differentially expressed in black skin in sheep and appears to influence melanosome formation by interacting with *mitf (Chen et al. 2016)*. In addition to *gpr143*, the gene *mitf* is upregulated in red skin patches, but the significance of this is unclear.

The melanocortin receptor 1 (*mc1r*) is probably the most well-known of all genes affecting variation in coloration and has been implicated in variation in melanic pigmentation in many vertebrates (e.g. Selz et al. 2007; Posso-Terranova and Andrés 2017). This gene was unexpectedly upregulated in both orange and red skin relative to black skin (Fig. 2a, b, d). In mammals, *mc1r* plays an important role in producing both dark skin and fur (via the production of eumelanin) and lighter, yellowish skin or fur (via the production of pheomelanin) (Swope and Abdel-Malek 2018). It is possible that *mc1r* plays a dual role in coloration in amphibians as well. However, this is speculative, as to date pheomelanin has only been identified in one species of frog, *Pachymedusa dacnicolor* (Wolnicka-Glubisz et al. 2012), and there is as-of-now no definitive evidence for *mc1r* causing a switch to pheomelanin production in amphibians. Recent research also indicates that the *mitf* gene, classically associated with melanogenesis, is also crucial for the development of iridophores (Petratou et al. 2018). This gene was found to be differentially expressed at various levels in the analyses presented here and in Stuckert et al. (2024).

### Iridophore/Guanine Platelet Pathways

Iridophores are a type of chromatophore found in ectotherms that contain structural elements (guanine platelet stacks) that reflect specific wavelengths of ambient light (Kelsh 2004). These have long been known to mediate the reflection of wavelengths in the green and blue part of the spectrum (Higdon et al. 2013). Recent research indicates that these elements also mediate variation in the yellow/orange and red part of the spectrum in poison frogs (and probably other taxa), due to their interactions with specific pigments (Twomey, et al. 2020a).

We found several genes involved in iridophore development to be differentially expressed between skin patches (e.g. *recql4, gart, ppat, paics, gart, impdh1*). Both *ppat* and *paics* were upregulated in either orange and/or red skin patches relative to black skin patches (Fig. 2a-b, d). The genes *paics* (phosphoribosylaminoimidazole carboxylase and phosphoribosylaminoimidazolesuccinocarboxamide synthase) and *ppat* (phosphoribosyl pyrophosphate amidotransferase) are in the purinosome group and play essential roles in purine biosynthesis (An et al. 2008). These genes are known to critically affect iridophore development and coloration in zebrafish, and mutations in *paics* in particular are known to cause extreme changes in coloration by dramatically reducing iridophore density in zebrafish (Ng et al. 2009). We note that this gene was also found to be differentially expressed between distinct color pattern morphs of a different poison frog, *D. auratus* (Stuckert et al. 2019).

The genes *impdh2, recql4,* and *gart* were found to be differentially expressed between orange skin patch from the Sauce morph and red skin patches from the Varadero morph (Fig. 2c). The *recql4* gene (RecQ-like helicase 4) showed differential expression between morphs of *R. imitator* (whole skin preps) (Stuckert et al. 2024) and was upregulated in orange skin patches of the Sauce morph relative to red skin patches of the Varadero morph. This gene is part of the Recq DNA helicase gene family, and mutations at this locus are associated with a variety of phenotypes in humans, including skin-related symptoms such as hypo- and hyper-pigmentation (Lu et al. 2017).

The results of our WGCNA/Cytoscape analyses implicated a central role for several genes associated with purine metabolism (*rab27a, impdh1, tfec, gmps*) (Fig. 3), which were upregulated in either orange, red, or orange/red skin patches relative to black skin patches (Fig. 2 a, b, d). The Rab GTPase 27a gene (*rab27a*) was upregulated in orange/red skin patches. Proteins from the Rab GTPase family are well-known membrane traffic regulators and may be involved in both melanosome transport and nucleotide exchange (Koike et al. 2019). Hence, the upregulation of *rab27a* may be associated with its role in purine synthesis. The *rab27a* locus produces a GTPase that is involved in guanine metabolism in various contexts (Figueiredo et al. 2008), which affects guanine platelet formation and iridophore development. The inosine monophosphate dehydrogenase 1 (*impdh1*) gene plays key roles in guanine synthesis and is associated with rate-limiting reaction in de novo biosynthesis of guanine nucleotides (Szekeres et al. 1992). Cleghorn et al. (2022) found that the loss of *impdh1* in zebrafish leads to the reduction of guanine levels.

In a recent investigation of the mechanisms underlying color pattern variation across *R. imitator* morphs and related species, Twomey, et al. (2020b) identified a strong association between variation guanine platelet shape (thickness) in iridophores and variation in spectral reflectance (color) across morphs and species. Hence, variation in the expression patterns of genes affecting guanine synthesis, platelet formation, and iridophore development may provide key insights into the genetic control of phenotypic variation between color morphs and between species.

### Pteridine Pathways

We identified several genes associated with the synthesis of pteridine to be differentially expressed between skin patches (*gch1, pax3-a, pax7, xdh*), all of which were upregulated in orange and/or red skin patches relative to black skin patches, consistent with their roles in colored pigment synthesis (Fig. 2a, b, d). Furthermore, these genes were all identified as hub genes in our WGCNA/Cytoscape analyses (Fig. 3). Evidence indicates that variation in pteridine pigment type and content was found to be significantly associated with variation in color (Twomey, et al. 2020b). Hence, the variation in the expression of these genes may provide further insights into the genetic control of coloration in these frogs.

The *xdh* (xanthine dehydrogenase) protein is a key hydroxylase in the purine oxidative metabolism pathway and well known to play crucial roles in the synthesis of pteridines, which are pigments deposited in xanthophores that play crucial roles in yellow, orange, and red coloration (Bagnara and Matsumoto 2006). This gene may be involved in both pteridine synthesis and the development of guanine platelets in iridophores. *Xdh* was found to be differentially expressed between morphs of *R. fantastica* in Stuckert et al. (2024). In our comparisons, we found these genes to be differentially expressed between black and orange/red skin patches in each morph independently and in the combined analysis. Stuckert et al. (2019) found that *xdh* is differentially expressed in different color pattern morphs of another poison frog, *D. auratus*. Variation in the expression of this gene may influence color variation through pathways affecting either the pteridine synthesis or the guanine platelet synthesis pathways. The *gch1* (GTP cyclohydrolase 1) locus codes for another enzyme that plays a key role in pteridine synthesis (Ziegler et al. 2000) and was differentially expressed in this study. *Gch1* is the rate-limiting enzyme in tetrahydrobiopterin (BH4) synthesis, which is a precursor to pteridine pigments. BH4 is a crucial cofactor for the enzyme phenylalanine hydroxylase, which metabolizes phenylalanine into tyrosine, and the enzyme tyrosine hydroxylase, which converts tyrosine to 3,4-dihydroxyphenylalanine (L-DOPA) (Storm and Kaufman 1968; O’Donnell et al. 1989; Wood et al. 1995; Nagatsu and Ichinose 1999). In fungi, the presence of L-DOPA results in the production of eumelanin (Figueiredo-Carvalho et al. 2014).

Members of the paired box 3/7 subfamily (*pax3* and *pax7*) showed differential expression between black and orange/red skin patches and (in the case of *pax3*) between different morphs of *R. fantastica* using whole skin preps (Stuckert et al. 2024). The transcriptional regulators paired box 3/7 (*pax3* and *pax7*) play roles in establishing functional xanthophore lineages during neural crest formation, with *pax3* in particular having a crucial influence on the development of pigmentation (Minchin and Hughes 2008). The paired box 3 (*pax3*) transcription factor is well known to play multiple roles in melanogenesis, including neural crest cell fate determination, differentiation, and melanocyte proliferation (Kubic et al. 2008; Medic and Ziman 2009). Previous research shows that *pax3* interacts with *mitf*, and with *sox10*, another key regulator of melanogenesis (Medic and Ziman 2009). Minchin and Hughes (2008) found that in zebrafish the loss of *Pax3* did not affect melanophores or iridophores, but instead resulted in defective specification of xanthophores. The *pax3* gene has strong effects on the development of precursors of both melanophores and xanthophores containing lighter colored pigments, which is potentially consistent with its higher expression in colored skin. The *pax7* transcription factor is essential for establishing and differentiating xanthophores in zebrafish (Nord et al. 2016). *Pax7* has also been identified as a key locus controlling color pattern variation in cichlid fish (Roberts et al. 2017).

### Carotenoid Metabolism

We identified several genes involved in carotenoid metabolism to be differentially expressed between skin patches, *akr1b1, dgat2,* and *rbp1*, all of which were upregulated in orange and/or red skin patches relative to black skin patches, consistent with their roles in colored pigment synthesis (Fig. 2a-b, d). These genes are all associated with the processing of retinoids and carotenoids, which are important for the development of pigmentation as well as many other developmental processes (Widjaja-Adhi and Golczak 2020). The diacylglycerol O-acyltransferase 2 (*dgat2*) gene encodes the acyltransferase enzyme and plays essential roles in the metabolic conversion of lipids and retinoids (Guo et al. 2009). The gene *dgat2* was upregulated in orange skin patches compared to black skin patches of the Sauce morph (Fig. 2a), but did not differ between red and black skin patches of the Varadero morph. The *dgat2* enzyme catalyzes the esterification of diacylglycerol and is associated with the production of yellow coloration in east African cichlid fish and yellow coloration on the throats of male Australian tawny dragon lizards (Ahi et al. 2020). Stuckert et al. (2024) found that *dgat2* was differentially expressed between larval developmental stages of *R. variabilis*.

### Synthesis

As described above, we have identified a number of genes that show differential expression between black and orange/red skin patches, as well as between the orange and red patches in the two morphs. The identification of these genes and their interaction networks is the first step in understanding their importance for the evolution of aposematic and mimetic coloration in an ecological and evolutionary framework. Moving forward, it will be important to manipulate key genes using molecular genetic techniques (e.g. CRISPR-Cas9) to pinpoint causal nodes and pathways. We acknowledge that, given that our analyses are restricted to embryos that are over a week old (and above), there may be patterns of differential gene expression that we have not detected, particularly those related to patterning. Practical considerations concerning the amounts of tissue required prevented us from analyzing gene expression at these stages, but technological progress (e.g. single-cell sequencing) will make it possible to investigate gene expression levels at very early embryonic stages in the future.

Our research does allow us to make some preliminary inferences concerning the relevance of our results to the evolution of coloration in these frogs, with potential relevance to other systems. Our results imply that the expression levels of a wide variety of different genes are involved in determining differences in color pattern between different mimetic morphs of *R. imitator*. Given that we are analyzing levels of expression, there is likely to be a smaller number of key genetic changes (likely in gene regulatory regions) that set off cascading effects of differential expression running through metabolic pathways and circuits. Identifying these key mutational changes will be an important goal moving forward.

### Conclusion

In this study, we investigated differential expression in comparisons between black and orange/red skin patches in two different morphs of the mimic poison frog (*R. imitator*), as well as between orange patches in the Sauce morph and red patches in the Varadero morph of this species. These analyses revealed the differential expression of multiple genes previously associated with color pattern development. In addition, we investigated coordinated patterns of coexpression by implementing a WGCNA on expression data between skin patches. These approaches allowed us to focus on genes that are likely to play central roles in color variation in *R. imitator* and hence in the developmental processes that allow this species to mimic a wide range of models across its native range in Peru. We found that a total of 184 unique genes from four major color-related pathways were differentially expressed between differently colored patches of skin (46 genes between black and yellow skin patches of the Sauce morph, 56 genes between black and red skin patches of the sauce morph, 116 genes between orange skin patches of the Sauce morph and the red skin patches of the Varadero morph, and 46 genes between black skin patches and orange/red skin patches of the Sauce and Varadero morph). This included genes such as *mc1r, slc24a5, mlana* and others known to play key roles in melanogenesis; genes such as *gmps, paics, ppat* and others known to play key role in guanine platelet synthesis and iridophore development; genes such as *xdh*, *recql4, pax7* and others known to play key roles in pteridine synthesis; and genes such as *akr1b1, dgat2, rbp1* and others known to play key roles in carotenoid metabolism.

## Materials and Methods

### Color Morphs

We established breeding colonies of the Sauce morph and the Varadero morph of *R. imitator* with frogs we obtained from Understory Enterprises, LLC. The color morphs we used for this project were captive bred individuals descended directly from wild populations with locality data from the north-central region of eastern Peru. Frogs captured in the wild were bred in captivity in a facility in Iquitos, Peru, and F1 offspring were exported to an additional breeding facility in Canada (this activity is carried out under permission from the Peruvian Ministry of Natural Resources), where offspring produced are made available for purchase to researchers and hobbyists. Although the bright color patterns of adults of the Sauce and Varadero morph are visually similar, reflectance values differ between these two morphs (Fig. 1b). We chose these two morphs because it is practical to effectively separate orange or red skin patches from black skin.

### Sample Collection

We kept established breeding pairs of *R. imitator* frogs of the same color morph in tanks with dimensions of 40.6 × 20.3 × 25.4 cm. We placed two polyvinyl chloride pipes filled with ∼ 25 mL of deionized water along the corners of each tank, which males used as breeding pools to deposit tadpoles. We checked tanks for new tadpoles three times a week. We collected tadpoles as soon as they were discovered in the breeding pool and individually reared them in 100 ml of deionized water. We performed full deionized water changes twice a week and fed tadpoles Omega One Marine Flakes fish food three times a week. We sacrificed froglets immediately after they reached the final stages of development, at Gosner stage 43 or 44 (Gosner 1960), by applying 20% benzocaine gel to their venter followed by pithing the cerebral region to ensure death. During these developmental stages, froglets had functional hind and forelimbs and displayed color and pattern elements reflecting their parental color morph. After froglets were euthanized, we dissected and separated different colored patches of the dorsal skin for each morph. We separated all orange skin from all black skin in the Sauce morph and all red skin from all black skin in the Varadero morph (Fig. 1a). Due to the limited amount of colored dorsal skin tissue on froglets and the minimal amount of RNA in skin, we combined all skin-colored patches from froglets bred from the same breeding pair to retrieve enough for RNA extraction (supplementary table S3, Supplementary Material online). Unfortunately, we were not able to ensure an equal representation of each individual as there was variation in sample quality. This yielded *N* = 5 per breeding pair, except one breeding pair from the Sauce morph and one breeding pair from the Varadero morph for which *N* = 1. We used a total of four families for the Varadero and Sauce morphs.

We separately stored each dissected skin color patch in RNAlater (Qiagen) at 4 °C for 24 h and then at −20 °C until RNA extraction. We then combined all skin patches dissected from tadpoles of the same breeding pairs and used a hybrid Trizol (Ambion) protocol to extract RNA, cleaned with DNAse and RNAsin, and purified with a RNeasy spin column (Qiagen) method. We verified RNA quality on a Bioanalyzer 2100 (Agilent) at the Genomic Core Facility (East Carolina University, NC, USA), and utilized samples with a RNA integrity number value >7. We sent samples to Novogene (Hong Kong, China), where double-stranded cDNA libraries were prepared and sequenced. Reads were paired end and 150 bp in length.

### Alignment and Read Counts

We used Trimmomatic version 0.36 to remove adaptors from our reads (Bolger et al. 2014). We then aligned our sequence data to a de novo assembled and annotated *R. imitator* genome (R_imi_1.0 (GCA_958301615.1)) (Stuckert et al. 2024) using the one-pass alignment procedure in STAR v2.7 (Dobin et al. 2013). When aligning reads, we allowed for a maximum of 20 multiple alignments per read and 10 base mismatches. The program htseq-count (Anders et al. 2015) was used to count gene expression from STAR alignments.

### Differential Gene Expression

We used the package DESeq2 (Love et al. 2014) to conduct all gene differential expression analyses in R version 4.3.2 “Eye Holes” (R Core Team 2023). We merged counts from htseq-count into a gene-level count in R by adding count data from transcripts that annotated to the same gene. We removed any genes that had a total experiment-wide expression level of ≤ 50, to avoid potential biases from genes with low expression.

We used a Wald's test with a Benjamini and Hochberg correction for multiple testing (Benjamini and Hochberg 1995) at an alpha value of 0.05 to identify differentially expressed genes. We conducted five different comparisons: two comparisons of skin patches within color morphs (Sauce: black vs. orange; Varadero: black vs. red), a comparison of the orange skin patches in the Sauce frogs to the red skin patches in the Varadero frogs, a comparison of the black skin patches in the Sauce frogs to the black skin patches in the Varadero frogs, and finally a comparison of skin patches between color morphs (all black skin patches vs. all color patches [orange and red]). For each model, we controlled for the paired nature of the data and general morph differences. We then identified and extracted genes associated with color pattern development (using a curated list of genes associated with coloration based on previous research (McLean et al. 2017; Baxter et al. 2019) from the differentially expressed genes found in our comparisons. Heatmaps were derived using the R package pheatmap (Kolde and Kolde 2015). We used the variance stabilized data of all genes to explore patterns of variation by conducting a PCA.

### Weighted Correlation Network Analysis

We conducted a WGCNA of our gene expression data to analyze networks of coexpressed genes across skin patches using the R package WGCNA version 1.6.8 (Langfelder and Horvath 2008). We used the variance stabilizing transformed data produced by DESeq2 to conduct our WGCNAs. Genes with total expression of ≤ 50 counts were excluded from the transformed dataset to avoid potential biases from genes with low expression. We identified gene network modules that correlated, Pearson’s correlation test, with the divergence between black and orange/red skin patches and between color morph skin patches at *P* < 0.05. We used a total of 16 skin patches for the gene network analysis (black [*N* = 8] versus orange/red [*N* = 8]). We used the scale-free topology criteria to determine the soft threshold (β) for both of our WGCNAs. We used β = 20 and a minimum module size of 30 when identifying networks that correlated with color differences, between black and orange/red skin patch, and color morph differences, Sauce and Varadero skin patch. We then identified the eigenmodules that were correlated with the phenotype categories at the *P*-value < 0.05.

### Hub Genes From the WGCNA Module Most Correlated with Color Differences and Color Morph Differences

Hub genes are those with high intra-module connectivity. Therefore, we extracted the top 50 ranked genes with the strongest connections from the most significant module, highest *r*-value (correlation between the grouping and module expression), with respect to the correlation with color differences, between black and orange/red skin patch, and color morph differences, Sauce and Varadero skin patch. After these hub genes were identified and extracted, we used the program Cytoscape to construct and visualize the network (Shannon et al. 2003).

## Supplementary Material

evae123_Supplementary_Data

## Data Availability

All raw read data are archived with the National Center for Biotechnology Information (accession number: PRJNA1121258). Codes for all analyses and gene expression counts are available on Zenodo (https://zenodo.org/records/11521755).
