# Supplementary material for: What Makes a Mimic? Orange, Red, and Black Color Production in the Mimic Poison Frog (Ranitomeya imitator)
Source: Genome Biol Evol. 2024 Jun 28;16(7):evae123. doi: 10.1093/gbe/evae123 (PMC11255871; doi:10.1093/gbe/evae123)
Supplement: evae123_Supplementary_Data [file evae123_supplementary_data.docx]

**Supplementary Tables**

**Table S1:** Number of differentially expressed genes from skin patch comparisons in *Ranitomeya imitator*.

| Skin color comparison | Significant genes (q<0.05) | Significant genes related to color production (q<0.05) | Percent of gene related to color production |
| --- | --- | --- | --- |
| Sauce: black vs orange | 278 | 46 | 16.54 % |
| Varadero: black vs red | 350 | 56 | 16 % |
| Sauce: orange vs Varadero: red | 1,499 | 116 | 7.73 % |
| Sauce: black vs Varadero: black | 1,353 | 97 | 7.16 % |
| All black vs orange/red) | 278 | 46 | 16.5*4* % |

**Table S2:** Differentially expressed genes between skin sections in *Ranitomeya imitator*. The first column indicates the name of genes found. The second column indicates the type of comparison. The third column indicates baseMean. The fourth column indicates log2FoldChange. The fifth column indicates lfcSE. The sixth column indicates stat. The seventh column indicates p-value. The eight column indicates adjusted P-value.Values for significance are all adjusted p-values.

| **Comparison** | **Gene** | **baseMean** | **log2FoldChange** | **lfcSE** | **stat** | **pvalue** | **padj** |
| --- | --- | --- | --- | --- | --- | --- | --- |
| Sauce: black vs orange | adgra2 | 142.886724 | -1.0086885 | 0.24006139 | -4.2017941 | 2.65E-05 | 0.00289505 |
| Sauce: black vs orange | arl6ip1 | 1970.6232 | 0.4953306 | 0.11904211 | 4.16096958 | 3.17E-05 | 0.00329831 |
| Sauce: black vs orange | clcn2 | 46.3313481 | 1.06626114 | 0.28966066 | 3.68106989 | 0.00023226 | 0.0151465 |
| Sauce: black vs orange | dio2 | 185.895161 | -0.6729893 | 0.18945847 | -3.5521732 | 0.00038206 | 0.02203098 |
| Sauce: black vs orange | dmxl2 | 83.3039636 | -0.7518031 | 0.22524928 | -3.3376494 | 0.0008449 | 0.03916818 |
| Sauce: black vs orange | erbb3 | 96.6365644 | -1.1107788 | 0.29039201 | -3.8251012 | 0.00013072 | 0.01008557 |
| Sauce: black vs orange | fscn2 | 29.9753067 | 1.42083954 | 0.33711766 | 4.21466961 | 2.50E-05 | 0.00276826 |
| Sauce: black vs orange | gmps | 730.405665 | 0.66298313 | 0.12884962 | 5.14540222 | 2.67E-07 | 5.73E-05 |
| Sauce: black vs orange | hmx1 | 61.9679746 | 1.29489137 | 0.26866471 | 4.81972999 | 1.44E-06 | 0.00024609 |
| Sauce: black vs orange | impdh1 | 394.87072 | 1.04460318 | 0.18440217 | 5.66480955 | 1.47E-08 | 4.24E-06 |
| Sauce: black vs orange | kcnj13 | 54.0342054 | -3.1259371 | 0.38097876 | -8.2050168 | 2.31E-16 | 1.68E-13 |
| Sauce: black vs orange | mab21l1 | 119.565948 | 1.21766552 | 0.20421442 | 5.96268135 | 2.48E-09 | 8.50E-07 |
| Sauce: black vs orange | mab21l2 | 44.2434161 | 2.12379127 | 0.31075432 | 6.83430977 | 8.24E-12 | 3.93E-09 |
| Sauce: black vs orange | mc1r | 695.088977 | 1.63119602 | 0.17680628 | 9.22589391 | 2.81E-20 | 2.37E-17 |
| Sauce: black vs orange | mlana | 1337.68064 | -1.5024393 | 0.13083679 | -11.483309 | 1.60E-30 | 1.95E-27 |
| Sauce: black vs orange | mlph | 415.804627 | 0.73651586 | 0.13474059 | 5.46617661 | 4.60E-08 | 1.17E-05 |
| Sauce: black vs orange | mreg | 77.6580157 | -1.4954934 | 0.22807671 | -6.5569754 | 5.49E-11 | 2.31E-08 |
| Sauce: black vs orange | otx1 | 325.521967 | 1.21223096 | 0.20594578 | 5.88616559 | 3.95E-09 | 1.31E-06 |
| Sauce: black vs orange | paics | 1979.98892 | 0.38553475 | 0.10450884 | 3.68901581 | 0.00022512 | 0.01485813 |
| Sauce: black vs orange | pax3-a | 76.1064196 | 2.61234988 | 0.25154612 | 10.3851728 | 2.90E-25 | 3.17E-22 |
| Sauce: black vs orange | pax7 | 31.7990118 | 4.36088887 | 0.55279702 | 7.88877058 | 3.05E-15 | 1.86E-12 |
| Sauce: black vs orange | pmel | 2597.18487 | -3.6023578 | 0.1114495 | -32.322781 | 3.35E-229 | 3.67E-225 |
| Sauce: black vs orange | ptch1 | 208.258041 | -0.7667805 | 0.19678788 | -3.8964823 | 9.76E-05 | 0.00848655 |
| Sauce: black vs orange | rab27a | 347.042108 | 0.61442533 | 0.13441282 | 4.5711811 | 4.85E-06 | 0.00072787 |
| Sauce: black vs orange | rbp1 | 327.167294 | 0.67443514 | 0.17000269 | 3.9672027 | 7.27E-05 | 0.00658457 |
| Sauce: black vs orange | rlbp1 | 105.121934 | 4.71474158 | 0.35227918 | 13.3835373 | 7.55E-41 | 1.18E-37 |
| Sauce: black vs orange | rpl27a | 17775.9955 | 0.48373452 | 0.14997583 | 3.22541645 | 0.0012579 | 0.04957375 |
| Sauce: black vs orange | rps7 | 18696.5273 | 0.51204374 | 0.15343683 | 3.33716317 | 0.00084638 | 0.03916818 |
| Sauce: black vs orange | scarb2 | 101.611549 | -0.8190749 | 0.22507529 | -3.6391149 | 0.00027358 | 0.01691867 |
| Sauce: black vs orange | slc16a2 | 31.4502033 | -1.801 | 0.45569687 | -3.9521886 | 7.74E-05 | 0.0068978 |
| Sauce: black vs orange | slc22a7 | 284.840013 | 5.66229508 | 0.27651344 | 20.477468 | 3.42E-93 | 1.25E-89 |
| Sauce: black vs orange | slc24a5 | 153.431089 | -1.0259873 | 0.16203557 | -6.3318644 | 2.42E-10 | 9.48E-08 |
| Sauce: black vs orange | slc45a2 | 76.606745 | -1.3369778 | 0.25783107 | -5.1854797 | 2.15E-07 | 4.82E-05 |
| Sauce: black vs orange | sox18 | 111.375606 | -0.8170614 | 0.24396257 | -3.3491262 | 0.00081067 | 0.03844886 |
| Sauce: black vs orange | tfec | 8.80024261 | 2.84899697 | 0.81110754 | 3.5124775 | 0.00044395 | 0.02440723 |
| Sauce: black vs orange | trpm1 | 221.099604 | 0.88265607 | 0.15915549 | 5.54587263 | 2.92E-08 | 7.82E-06 |
| Sauce: black vs orange | trpm7 | 125.849399 | -0.8278764 | 0.20606049 | -4.0176378 | 5.88E-05 | 0.0055898 |
| Sauce: black vs orange | tyrp1 | 585.635233 | -4.3961237 | 0.19128277 | -22.982329 | 7.00E-117 | 3.84E-113 |
| Sauce: black vs orange | usp43 | 156.166748 | -0.7349894 | 0.19806338 | -3.7108798 | 0.00020654 | 0.01414284 |
| Sauce: black vs orange | dapl1-a | 1590.02025 | 0.6657077 | 0.17281769 | 3.85208078 | 0.00011712 | 0.00950481 |
| Sauce: black vs orange | pax3-a | 76.1064196 | 2.61234988 | 0.25154612 | 10.3851728 | 2.90E-25 | 3.17E-22 |
| Sauce: black vs orange | tmem159-a | 201.028125 | 0.55132716 | 0.16825703 | 3.27669606 | 0.00105029 | 0.04413988 |
| Sauce: black vs orange | gch1 | 3744.25712 | 5.52331694 | 0.35969332 | 15.3556282 | 3.25E-53 | 5.93E-50 |
| Sauce: black vs orange | xdh | 1063.61991 | 4.61034869 | 0.27218073 | 16.9385565 | 2.34E-64 | 6.40E-61 |
| Sauce: black vs orange | cyp26b1 | 109.033588 | -0.8834912 | 0.27036024 | -3.2678297 | 0.00108376 | 0.04413988 |
| Sauce: black vs orange | dgat2 | 128.879338 | 0.65564112 | 0.19176156 | 3.41904356 | 0.00062842 | 0.03115354 |
| Varadero: black vs red | adgra2 | 142.8867241 | -1.101074115 | 0.241547506 | -4.558416411 | 5.15E-06 | 0.000464324 |
| Varadero: black vs red | arl6ip1 | 1970.623202 | 0.515421523 | 0.119143087 | 4.326071583 | 1.52E-05 | 0.001153517 |
| Varadero: black vs red | cbs | 406.9101254 | 0.440781311 | 0.129904226 | 3.393125263 | 0.000691 | 0.027372924 |
| Varadero: black vs red | col18a1 | 2421.430436 | -0.407252973 | 0.094133242 | -4.326345998 | 1.52E-05 | 0.001153517 |
| Varadero: black vs red | dio2 | 185.8951611 | -0.820037736 | 0.195784492 | -4.188471357 | 2.81E-05 | 0.00197312 |
| Varadero: black vs red | en1-b | 45.08923615 | -1.17511875 | 0.341096379 | -3.445122321 | 0.000570801 | 0.023816654 |
| Varadero: black vs red | fhl1 | 390.9368049 | -0.583709582 | 0.166561929 | -3.504459793 | 0.000457535 | 0.020692792 |
| Varadero: black vs red | flna | 8547.618258 | -0.343903272 | 0.09118058 | -3.771672326 | 0.000162157 | 0.008708928 |
| Varadero: black vs red | fscn2 | 29.97530673 | 1.336855386 | 0.362988509 | 3.682913793 | 0.000230583 | 0.011499305 |
| Varadero: black vs red | gpr143 | 148.2680366 | 0.808685899 | 0.206702115 | 3.912325227 | 9.14E-05 | 0.005374526 |
| Varadero: black vs red | hmx1 | 61.96797458 | 1.577206808 | 0.286572719 | 5.503687914 | 3.72E-08 | 5.84E-06 |
| Varadero: black vs red | hoxb7-b | 20.39801298 | -3.527869829 | 0.557414114 | -6.328992644 | 2.47E-10 | 5.30E-08 |
| Varadero: black vs red | impdh1 | 394.8707204 | 1.106254112 | 0.18509629 | 5.976641207 | 2.28E-09 | 4.39E-07 |
| Varadero: black vs red | kcnj13 | 54.03420539 | -2.215083712 | 0.323738424 | -6.84220207 | 7.80E-12 | 2.18E-09 |
| Varadero: black vs red | krt1 | 234635.6543 | 0.331142028 | 0.086545584 | 3.826215173 | 0.000130129 | 0.007196367 |
| Varadero: black vs red | lama1 | 354.1336808 | -0.429371239 | 0.127946649 | -3.35586154 | 0.000791182 | 0.03071241 |
| Varadero: black vs red | lmx1b.1 | 364.9544399 | 0.59681432 | 0.144664541 | 4.125505222 | 3.70E-05 | 0.002519754 |
| Varadero: black vs red | lrp5 | 346.176046 | -0.500851897 | 0.139170835 | -3.598827994 | 0.000319655 | 0.015325582 |
| Varadero: black vs red | lvrn | 157.9500555 | -0.625686639 | 0.175819194 | -3.558693602 | 0.000372704 | 0.017347819 |
| Varadero: black vs red | mab21l1 | 119.5659475 | 1.797363836 | 0.215781208 | 8.329566116 | 8.11E-17 | 3.94E-14 |
| Varadero: black vs red | mab21l2 | 44.24341606 | 4.611260308 | 0.58905947 | 7.828174481 | 4.95E-15 | 2.05E-12 |
| Varadero: black vs red | mc1r | 695.0889768 | 1.731476241 | 0.176710411 | 9.798382743 | 1.14E-22 | 7.52E-20 |
| Varadero: black vs red | mitf | 60.08457609 | 1.065225134 | 0.255139719 | 4.175065874 | 2.98E-05 | 0.002079899 |
| Varadero: black vs red | mlana | 1337.680639 | -0.738700718 | 0.128749137 | -5.737519755 | 9.61E-09 | 1.73E-06 |
| Varadero: black vs red | mlph | 415.8046266 | 0.426949162 | 0.128783625 | 3.315244163 | 0.000915631 | 0.033869255 |
| Varadero: black vs red | oat | 635.942316 | 0.451157124 | 0.135393676 | 3.332187566 | 0.000861662 | 0.032964458 |
| Varadero: black vs red | otx1 | 325.5219672 | 2.053173291 | 0.203430105 | 10.09277017 | 5.95E-24 | 4.15E-21 |
| Varadero: black vs red | otx2 | 5.685883171 | 3.975352488 | 1.07322182 | 3.704129394 | 0.000212118 | 0.010937375 |
| Varadero: black vs red | pax3-a | 76.10641965 | 3.34912616 | 0.288307945 | 11.6164893 | 3.40E-31 | 3.45E-28 |
| Varadero: black vs red | pax6 | 34.39841939 | 6.733999418 | 1.174259569 | 5.734677065 | 9.77E-09 | 1.73E-06 |
| Varadero: black vs red | pax7 | 31.79901178 | 3.498303076 | 0.493231798 | 7.092614651 | 1.32E-12 | 4.20E-10 |
| Varadero: black vs red | pcdh10 | 344.426113 | 0.583714928 | 0.146287228 | 3.990197466 | 6.60E-05 | 0.004074533 |
| Varadero: black vs red | pde6a | 22.50406563 | 2.863212902 | 0.666517039 | 4.295783506 | 1.74E-05 | 0.001305113 |
| Varadero: black vs red | pmel | 2597.184873 | -2.617242907 | 0.106531982 | -24.56767309 | 2.80E-133 | 3.13E-129 |
| Varadero: black vs red | pxdn | 841.0822609 | -0.341427438 | 0.104084485 | -3.280291365 | 0.001036999 | 0.037356593 |
| Varadero: black vs red | rab27a | 347.0421081 | 0.669282112 | 0.135891346 | 4.92512683 | 8.43E-07 | 9.06E-05 |
| Varadero: black vs red | rbp1 | 327.1672935 | 0.952994813 | 0.177506661 | 5.368783381 | 7.93E-08 | 1.15E-05 |
| Varadero: black vs red | rlbp1 | 105.1219335 | 4.324264142 | 0.359088969 | 12.04231962 | 2.13E-33 | 2.38E-30 |
| Varadero: black vs red | six3 | 9.870572553 | 6.958468776 | 1.221831686 | 5.69511239 | 1.23E-08 | 2.15E-06 |
| Varadero: black vs red | slc22a7 | 284.8400126 | 5.182899375 | 0.25593927 | 20.25050464 | 3.52E-91 | 1.31E-87 |
| Varadero: black vs red | slc24a5 | 153.4310886 | -1.116247194 | 0.163627685 | -6.821872435 | 8.99E-12 | 2.45E-09 |
| Varadero: black vs red | slc45a2 | 76.60674496 | -1.179323803 | 0.249228862 | -4.731890976 | 2.22E-06 | 0.000223861 |
| Varadero: black vs red | sox18 | 111.3756057 | -0.839637793 | 0.244428777 | -3.435102051 | 0.000592331 | 0.024416703 |
| Varadero: black vs red | trpm1 | 221.0996035 | 0.694611994 | 0.158104538 | 4.393371633 | 1.12E-05 | 0.000890537 |
| Varadero: black vs red | tyrp1 | 585.6352333 | -2.520306165 | 0.171730268 | -14.67595781 | 9.19E-49 | 1.71E-45 |
| Varadero: black vs red | wnt7a | 352.8220174 | -0.465047345 | 0.132599756 | -3.507150826 | 0.000452932 | 0.020567916 |
| Varadero: black vs red | zic1 | 195.8949247 | -1.368841691 | 0.250042341 | -5.474439596 | 4.39E-08 | 6.63E-06 |
| Varadero: black vs red | esyt2-a | 215.4040579 | -0.730557347 | 0.220506117 | -3.313093337 | 0.000922702 | 0.034018158 |
| Varadero: black vs red | hsd11b1l-a | 2261.73133 | 0.584335637 | 0.161912139 | 3.608967438 | 0.000307418 | 0.014802451 |
| Varadero: black vs red | odc1-a | 1812.153958 | 0.320656429 | 0.096492293 | 3.323129948 | 0.000890134 | 0.033145637 |
| Varadero: black vs red | pax3-a | 76.10641965 | 3.34912616 | 0.288307945 | 11.6164893 | 3.40E-31 | 3.45E-28 |
| Varadero: black vs red | wnt2b-a | 10.35634497 | -2.854362158 | 0.690789055 | -4.132031532 | 3.60E-05 | 0.002464275 |
| Varadero: black vs red | gch1 | 3744.25712 | 5.476664361 | 0.359308757 | 15.24222345 | 1.85E-52 | 4.14E-49 |
| Varadero: black vs red | xdh | 1063.619912 | 7.046614499 | 0.328405139 | 21.45707742 | 3.92E-102 | 2.19E-98 |
| Varadero: black vs red | rbp2 | 30.95335526 | 1.348714464 | 0.355131249 | 3.797791569 | 0.000145991 | 0.007955441 |
| Varadero: black vs red | wnt2b-a | 10.35634497 | -2.854362158 | 0.690789055 | -4.132031532 | 3.60E-05 | 0.002464275 |
| Sauce orange vs Varadero red | abca12 | 68.69887072 | 1.267286978 | 0.399606603 | 3.171336428 | 0.001517393 | 0.015279924 |
| Sauce orange vs Varadero red | abca4 | 63.74126944 | 2.460413208 | 0.416716157 | 5.904290411 | 3.54E-09 | 1.72E-07 |
| Sauce orange vs Varadero red | adam9 | 586.9742393 | 0.552881016 | 0.194046059 | 2.849225687 | 0.004382578 | 0.035227824 |
| Sauce orange vs Varadero red | aldoa | 5083.300006 | -0.835462159 | 0.165346519 | -5.052795554 | 4.35E-07 | 1.36E-05 |
| Sauce orange vs Varadero red | alkbh1 | 551.4319212 | 0.699906527 | 0.204519755 | 3.422195218 | 0.000621177 | 0.007503434 |
| Sauce orange vs Varadero red | arhgef18 | 29.03904777 | 1.796120918 | 0.611143989 | 2.938948842 | 0.003293274 | 0.028298912 |
| Sauce orange vs Varadero red | atox1 | 1467.824598 | -0.660548508 | 0.220420055 | -2.996771356 | 0.002728553 | 0.024284341 |
| Sauce orange vs Varadero red | bbs1 | 174.9294836 | -1.121260601 | 0.254134229 | -4.412080195 | 1.02E-05 | 0.000225241 |
| Sauce orange vs Varadero red | bbs5 | 102.3126151 | -1.225932187 | 0.370305459 | -3.310597121 | 0.000930971 | 0.010334065 |
| Sauce orange vs Varadero red | blm | 186.6985228 | -0.91220848 | 0.248297642 | -3.67385075 | 0.000238922 | 0.003422859 |
| Sauce orange vs Varadero red | bsg | 3274.346933 | -0.491510398 | 0.171780757 | -2.861265761 | 0.004219532 | 0.034115732 |
| Sauce orange vs Varadero red | cars | 1453.790617 | -0.455265792 | 0.157016729 | -2.899473171 | 0.003737903 | 0.031095269 |
| Sauce orange vs Varadero red | cbs | 406.9101254 | 0.704663734 | 0.206363988 | 3.414664255 | 0.000638607 | 0.007680533 |
| Sauce orange vs Varadero red | cdc42 | 1831.189358 | -0.475603672 | 0.15560166 | -3.056546264 | 0.002239029 | 0.020824112 |
| Sauce orange vs Varadero red | cdkn1b | 389.1087839 | 1.114691206 | 0.236685817 | 4.709581746 | 2.48E-06 | 6.40E-05 |
| Sauce orange vs Varadero red | cdkn2a | 196.6401445 | 4.098114798 | 0.349786975 | 11.71603032 | 1.06E-31 | 6.08E-29 |
| Sauce orange vs Varadero red | clcn7 | 354.7123175 | 0.971387252 | 0.199317134 | 4.873576246 | 1.10E-06 | 3.13E-05 |
| Sauce orange vs Varadero red | col17a1 | 8730.205381 | -0.59819271 | 0.12661977 | -4.724323151 | 2.31E-06 | 6.01E-05 |
| Sauce orange vs Varadero red | cpsf1 | 1007.727525 | 0.533958883 | 0.164687462 | 3.242255823 | 0.001185875 | 0.012553091 |
| Sauce orange vs Varadero red | csnk1a1 | 2113.812845 | 0.469480614 | 0.140263053 | 3.347143855 | 0.000816488 | 0.009347383 |
| Sauce orange vs Varadero red | ctc1 | 280.8555733 | 0.588787403 | 0.216020207 | 2.725612619 | 0.006418228 | 0.047608736 |
| Sauce orange vs Varadero red | ctns | 301.924659 | 0.794811084 | 0.209871293 | 3.787135779 | 0.000152394 | 0.002290297 |
| Sauce orange vs Varadero red | dct | 4037.664766 | 0.931375457 | 0.189644882 | 4.911155245 | 9.05E-07 | 2.64E-05 |
| Sauce orange vs Varadero red | dnm2 | 1847.350012 | 0.440820139 | 0.141067466 | 3.124888762 | 0.001778723 | 0.017462088 |
| Sauce orange vs Varadero red | dsg4 | 108.3120123 | 1.428776945 | 0.459738109 | 3.107806201 | 0.001884816 | 0.018339295 |
| Sauce orange vs Varadero red | en1-b | 45.08923615 | -1.505896652 | 0.525738216 | -2.864346947 | 0.0041787 | 0.033837281 |
| Sauce orange vs Varadero red | ercc3 | 1171.021437 | -0.500495613 | 0.164336517 | -3.045553246 | 0.002322527 | 0.021329087 |
| Sauce orange vs Varadero red | fanca | 454.3875025 | -0.522153986 | 0.185933543 | -2.808282885 | 0.004980645 | 0.038893851 |
| Sauce orange vs Varadero red | fat1 | 658.9597919 | -0.57970081 | 0.19068023 | -3.040172597 | 0.002364426 | 0.021615027 |
| Sauce orange vs Varadero red | gart | 509.5060558 | 0.630866626 | 0.182518741 | 3.456448489 | 0.000547344 | 0.006822183 |
| Sauce orange vs Varadero red | gfpt1 | 372.7765557 | 0.961870736 | 0.216885826 | 4.434917467 | 9.21E-06 | 0.000206229 |
| Sauce orange vs Varadero red | gja5 | 361.5906172 | -0.617325406 | 0.210239516 | -2.936295793 | 0.003321574 | 0.028475093 |
| Sauce orange vs Varadero red | gnas | 186.1328318 | 0.895873604 | 0.265792227 | 3.370578647 | 0.000750105 | 0.008761353 |
| Sauce orange vs Varadero red | gtpbp3 | 48.71378224 | -1.387798926 | 0.463789666 | -2.99230239 | 0.002768819 | 0.024543023 |
| Sauce orange vs Varadero red | hdac2 | 635.9642683 | -0.602542386 | 0.211939431 | -2.842993314 | 0.004469201 | 0.035688458 |
| Sauce orange vs Varadero red | hoxb7-b | 20.39801298 | -2.188914458 | 0.770404907 | -2.841251969 | 0.004493679 | 0.035857793 |
| Sauce orange vs Varadero red | hps1 | 225.1434415 | 0.663824035 | 0.233384581 | 2.844335438 | 0.004450417 | 0.035564381 |
| Sauce orange vs Varadero red | ift27 | 200.4358597 | -0.862041992 | 0.241752981 | -3.565796735 | 0.000362752 | 0.004912628 |
| Sauce orange vs Varadero red | ikbkb | 871.1483097 | 0.482709966 | 0.153748405 | 3.13960959 | 0.001691731 | 0.016727985 |
| Sauce orange vs Varadero red | kcnj13 | 54.03420539 | 1.980600191 | 0.57261219 | 3.45888583 | 0.000542415 | 0.006776164 |
| Sauce orange vs Varadero red | krt1 | 234635.6543 | 1.526652541 | 0.136862708 | 11.15462763 | 6.80E-29 | 3.55E-26 |
| Sauce orange vs Varadero red | lrat | 62.37661011 | 1.574265731 | 0.404692105 | 3.890033212 | 0.000100231 | 0.001586886 |
| Sauce orange vs Varadero red | mcm2 | 1117.736494 | -0.624872468 | 0.167819794 | -3.723472986 | 0.000196501 | 0.002882013 |
| Sauce orange vs Varadero red | meox1 | 116.3674229 | -0.953220097 | 0.291840075 | -3.266241268 | 0.001089854 | 0.011740841 |
| Sauce orange vs Varadero red | mfsd2a | 91.06556959 | 1.180876352 | 0.357499003 | 3.303159849 | 0.000956019 | 0.010547977 |
| Sauce orange vs Varadero red | mgrn1 | 1308.357403 | 0.396770935 | 0.144609053 | 2.743748927 | 0.006074198 | 0.045550245 |
| Sauce orange vs Varadero red | mlana | 1337.680639 | 0.714913887 | 0.206596199 | 3.460440652 | 0.000539292 | 0.006761013 |
| Sauce orange vs Varadero red | mlph | 415.8046266 | 0.834816231 | 0.207124542 | 4.03050369 | 5.57E-05 | 0.000981937 |
| Sauce orange vs Varadero red | mmp17 | 94.05660628 | -1.004684458 | 0.341251404 | -2.944118165 | 0.003238763 | 0.027901747 |
| Sauce orange vs Varadero red | mpnd | 106.2067942 | -1.88727363 | 0.336143183 | -5.614493239 | 1.97E-08 | 8.24E-07 |
| Sauce orange vs Varadero red | mpp5 | 183.683597 | 0.912843886 | 0.317616673 | 2.874042721 | 0.004052541 | 0.033035449 |
| Sauce orange vs Varadero red | mpv17 | 186.1242257 | -0.939429208 | 0.276472164 | -3.397916071 | 0.000679012 | 0.008078969 |
| Sauce orange vs Varadero red | mpzl3 | 118.186085 | 1.376744142 | 0.306039309 | 4.498585964 | 6.84E-06 | 0.000158496 |
| Sauce orange vs Varadero red | nmnat1 | 64.59531307 | 1.71807189 | 0.416472871 | 4.125291253 | 3.70E-05 | 0.000689904 |
| Sauce orange vs Varadero red | oat | 635.942316 | 0.702171304 | 0.21488091 | 3.267723052 | 0.001084164 | 0.011702562 |
| Sauce orange vs Varadero red | obscn | 11.05606695 | 6.49185633 | 1.827048175 | 3.553193845 | 0.000380584 | 0.005122454 |
| Sauce orange vs Varadero red | otx1 | 325.5219672 | 1.380597601 | 0.319650614 | 4.319083217 | 1.57E-05 | 0.000330746 |
| Sauce orange vs Varadero red | ovol1 | 254.6136139 | 0.73941259 | 0.22703315 | 3.256848573 | 0.001126565 | 0.01201907 |
| Sauce orange vs Varadero red | phactr4-b | 909.0556906 | 0.710011969 | 0.156338927 | 4.541491898 | 5.59E-06 | 0.00013379 |
| Sauce orange vs Varadero red | pmel | 2597.184873 | 1.304130602 | 0.175369722 | 7.436463871 | 1.03E-13 | 1.09E-11 |
| Sauce orange vs Varadero red | pomgnt1 | 512.8482364 | 0.542245794 | 0.174000299 | 3.116349791 | 0.001831049 | 0.017902421 |
| Sauce orange vs Varadero red | prdm1 | 311.4929487 | 1.409464566 | 0.289714124 | 4.865018478 | 1.14E-06 | 3.25E-05 |
| Sauce orange vs Varadero red | prkci | 324.5975646 | 1.078245066 | 0.250620308 | 4.302305246 | 1.69E-05 | 0.000354091 |
| Sauce orange vs Varadero red | ptpn6 | 314.4708734 | -1.129945924 | 0.220887264 | -5.115486991 | 3.13E-07 | 1.00E-05 |
| Sauce orange vs Varadero red | pxdn | 841.0822609 | -0.493626218 | 0.163010219 | -3.028191851 | 0.002460218 | 0.022308959 |
| Sauce orange vs Varadero red | rabggta | 439.0101636 | -1.695430891 | 0.196129034 | -8.644466651 | 5.41E-18 | 9.44E-16 |
| Sauce orange vs Varadero red | raf1 | 444.1020591 | 0.62688637 | 0.215423039 | 2.91002472 | 0.003614002 | 0.030225193 |
| Sauce orange vs Varadero red | raph1 | 715.7977352 | -0.638910808 | 0.162738452 | -3.925997814 | 8.64E-05 | 0.001408155 |
| Sauce orange vs Varadero red | recql4 | 127.5209307 | -1.475699572 | 0.298121819 | -4.949988492 | 7.42E-07 | 2.22E-05 |
| Sauce orange vs Varadero red | rho | 39.28872701 | -1.427955942 | 0.479628522 | -2.977212312 | 0.002908825 | 0.025536127 |
| Sauce orange vs Varadero red | rp2 | 25.1203528 | 3.195785142 | 0.710064806 | 4.500695029 | 6.77E-06 | 0.000157705 |
| Sauce orange vs Varadero red | rpe65 | 166.894072 | 1.113775113 | 0.284751104 | 3.911398747 | 9.18E-05 | 0.001478466 |
| Sauce orange vs Varadero red | sdc4-b | 2096.807067 | 0.497516772 | 0.143275972 | 3.472436899 | 0.000515756 | 0.006540076 |
| Sauce orange vs Varadero red | sh3pxd2a | 67.73263071 | -1.125994744 | 0.410664951 | -2.741881773 | 0.006108832 | 0.045716099 |
| Sauce orange vs Varadero red | slc12a2 | 935.8476794 | -0.744719604 | 0.185587578 | -4.012766439 | 6.00E-05 | 0.001041971 |
| Sauce orange vs Varadero red | slc2a1 | 20.17195844 | 4.395369469 | 0.930036256 | 4.726019488 | 2.29E-06 | 5.99E-05 |
| Sauce orange vs Varadero red | slc30a4 | 89.02877151 | 1.203888843 | 0.337383575 | 3.568308987 | 0.000359293 | 0.004877832 |
| Sauce orange vs Varadero red | slc31a1 | 807.4401786 | -0.810951167 | 0.168033167 | -4.826137485 | 1.39E-06 | 3.85E-05 |
| Sauce orange vs Varadero red | sytl2 | 1241.532318 | 0.726461807 | 0.167258496 | 4.343347721 | 1.40E-05 | 0.00029853 |
| Sauce orange vs Varadero red | tbc1d32 | 39.65761733 | -1.412132399 | 0.497072228 | -2.840899811 | 0.004498644 | 0.035871286 |
| Sauce orange vs Varadero red | terf2 | 367.1522718 | 1.559744344 | 0.218172759 | 7.149125087 | 8.73E-13 | 7.84E-11 |
| Sauce orange vs Varadero red | tgfbr2 | 154.818907 | 1.610159695 | 0.293021487 | 5.495022598 | 3.91E-08 | 1.55E-06 |
| Sauce orange vs Varadero red | tshr | 102.7734306 | -3.101507451 | 0.41742276 | -7.430134977 | 1.08E-13 | 1.13E-11 |
| Sauce orange vs Varadero red | tspan36 | 486.1981774 | -0.959004244 | 0.253330393 | -3.785587004 | 0.000153346 | 0.002298305 |
| Sauce orange vs Varadero red | ttc8 | 119.2344053 | 1.41814706 | 0.345769344 | 4.101425082 | 4.11E-05 | 0.000757347 |
| Sauce orange vs Varadero red | tub | 142.0667149 | -0.82856169 | 0.268035806 | -3.09123509 | 0.001993258 | 0.019122705 |
| Sauce orange vs Varadero red | tyms | 350.5669109 | -0.615189033 | 0.224810356 | -2.736479958 | 0.006210038 | 0.046283792 |
| Sauce orange vs Varadero red | tyr | 2363.236727 | 0.930375768 | 0.155886079 | 5.96830566 | 2.40E-09 | 1.24E-07 |
| Sauce orange vs Varadero red | tyrp1 | 585.6352333 | 1.82485589 | 0.296979608 | 6.144717815 | 8.01E-10 | 4.69E-08 |
| Sauce orange vs Varadero red | vldlr | 396.6232477 | -0.528578984 | 0.194262816 | -2.720947808 | 0.006509504 | 0.048036508 |
| Sauce orange vs Varadero red | wrap53 | 652.3854115 | -0.62238532 | 0.202652528 | -3.07119445 | 0.002132043 | 0.020182907 |
| Sauce orange vs Varadero red | wrn | 196.527755 | -0.712422706 | 0.235278531 | -3.027997084 | 0.002461805 | 0.022308959 |
| Sauce orange vs Varadero red | ywhaz | 5354.424291 | 0.464025825 | 0.134822978 | 3.441741396 | 0.000577983 | 0.007111473 |
| Sauce orange vs Varadero red | zic1 | 195.8949247 | -2.369254093 | 0.389301432 | -6.085911576 | 1.16E-09 | 6.54E-08 |
| Sauce orange vs Varadero red | znf503 | 181.6340245 | -0.820483774 | 0.262510714 | -3.1255249 | 0.00177488 | 0.017439991 |
| Sauce orange vs Varadero red | aktip-a | 895.565515 | 0.501900666 | 0.173444519 | 2.893724577 | 0.003807019 | 0.031384277 |
| Sauce orange vs Varadero red | daam1-a | 60.98729904 | 2.483125141 | 0.540005032 | 4.598337045 | 4.26E-06 | 0.000103918 |
| Sauce orange vs Varadero red | dbnl-a | 71.08109129 | 1.989543801 | 0.451182507 | 4.409620878 | 1.04E-05 | 0.000226903 |
| Sauce orange vs Varadero red | dusp1-a | 1589.565975 | 0.606217135 | 0.172895888 | 3.506255358 | 0.000454459 | 0.005913364 |
| Sauce orange vs Varadero red | fcn1-a | 255.8924228 | -2.16871501 | 0.27108053 | -8.000261075 | 1.24E-15 | 1.64E-13 |
| Sauce orange vs Varadero red | irs2-a | 403.1238298 | 1.237473067 | 0.253827445 | 4.875253209 | 1.09E-06 | 3.11E-05 |
| Sauce orange vs Varadero red | kdm3a-a | 1890.051962 | -0.588133776 | 0.171814273 | -3.423078679 | 0.000619162 | 0.007487344 |
| Sauce orange vs Varadero red | odc1-a | 1812.153958 | 0.422428099 | 0.152496633 | 2.77008148 | 0.005604227 | 0.042906997 |
| Sauce orange vs Varadero red | ralbp1-a | 406.075971 | 0.842536309 | 0.220118027 | 3.827657007 | 0.000129369 | 0.001990682 |
| Sauce orange vs Varadero red | rasgrp2-a | 45.45772427 | -1.634975197 | 0.527190438 | -3.101299034 | 0.001926736 | 0.01864781 |
| Sauce orange vs Varadero red | slc22a6-a | 61.20075418 | 2.286447548 | 0.460817614 | 4.961719084 | 6.99E-07 | 2.12E-05 |
| Sauce orange vs Varadero red | tlcd4-a | 622.1618987 | 0.974275971 | 0.195251273 | 4.989857202 | 6.04E-07 | 1.85E-05 |
| Sauce orange vs Varadero red | gchfr | 46.82704635 | -4.009052824 | 0.592695015 | -6.764107551 | 1.34E-11 | 1.06E-09 |
| Sauce orange vs Varadero red | rbp2 | 30.95335526 | 9.352089546 | 1.632543359 | 5.728539761 | 1.01E-08 | 4.49E-07 |
| Sauce orange vs Varadero red | rbp4 | 29138.73354 | -0.543481543 | 0.173230515 | -3.137331456 | 0.001704933 | 0.016800743 |
| Sauce orange vs Varadero red | rdh14 | 1039.406708 | -0.712299864 | 0.220112581 | -3.236070652 | 0.001211874 | 0.012803558 |
| Sauce orange vs Varadero red | rxrb | 556.2372063 | 0.71110477 | 0.187598004 | 3.79057748 | 0.000150297 | 0.002268125 |
| Sauce orange vs Varadero red | sult1a1 | 298.0784565 | -2.714732453 | 0.307271441 | -8.834965099 | 1.00E-18 | 2.03E-16 |
| Sauce orange vs Varadero red | adcy7 | 150.2393459 | 1.859564201 | 0.310230654 | 5.994134282 | 2.05E-09 | 1.09E-07 |
| Sauce orange vs Varadero red | creb3l1 | 1681.862788 | -0.966414704 | 0.169382526 | -5.70551594 | 1.16E-08 | 5.08E-07 |
| Sauce orange vs Varadero red | dvl1 | 123.7581216 | 1.141115943 | 0.355773049 | 3.207426603 | 0.001339282 | 0.013816551 |
| Sauce black vs Varadero black | abca12 | 68.69887072 | 1.188937409 | 0.394811221 | 3.011407342 | 0.002600398 | 0.026241229 |
| Sauce black vs Varadero black | abca4 | 63.74126944 | 2.659248195 | 0.429848846 | 6.186472797 | 6.15E-10 | 4.43E-08 |
| Sauce black vs Varadero black | aldoa | 5083.300006 | -0.906336265 | 0.165396038 | -5.479794297 | 4.26E-08 | 2.08E-06 |
| Sauce black vs Varadero black | alkbh1 | 551.4319212 | 0.698392417 | 0.204833041 | 3.409569153 | 0.000650656 | 0.008632394 |
| Sauce black vs Varadero black | atox1 | 1467.824598 | -0.661188943 | 0.220647589 | -2.99658358 | 0.002730234 | 0.027134735 |
| Sauce black vs Varadero black | bbs1 | 174.9294836 | -1.089525063 | 0.253570673 | -4.296731361 | 1.73E-05 | 0.000411984 |
| Sauce black vs Varadero black | bbs5 | 102.3126151 | -1.279021658 | 0.369740636 | -3.459240166 | 0.000541702 | 0.007406792 |
| Sauce black vs Varadero black | blm | 186.6985228 | -0.763965413 | 0.246648104 | -3.097390164 | 0.001952327 | 0.020970623 |
| Sauce black vs Varadero black | cdkn1b | 389.1087839 | 0.886111895 | 0.236432818 | 3.747837976 | 0.000178365 | 0.003032755 |
| Sauce black vs Varadero black | cdkn2a | 196.6401445 | 3.390136868 | 0.345198376 | 9.820836656 | 9.16E-23 | 4.09E-20 |
| Sauce black vs Varadero black | clcn7 | 354.7123175 | 1.128759002 | 0.19945531 | 5.659207573 | 1.52E-08 | 8.33E-07 |
| Sauce black vs Varadero black | col17a1 | 8730.205381 | -0.742334268 | 0.126628336 | -5.862307687 | 4.56E-09 | 2.93E-07 |
| Sauce black vs Varadero black | cpsf1 | 1007.727525 | 0.683490502 | 0.164673573 | 4.150577963 | 3.32E-05 | 0.000738456 |
| Sauce black vs Varadero black | csnk1a1 | 2113.812845 | 0.393089457 | 0.140328336 | 2.801212283 | 0.005091102 | 0.043741702 |
| Sauce black vs Varadero black | ctc1 | 280.8555733 | 0.628024206 | 0.215373821 | 2.915972802 | 0.003545814 | 0.033079786 |
| Sauce black vs Varadero black | ctns | 301.924659 | 0.698488211 | 0.209090869 | 3.340596433 | 0.000835986 | 0.01064858 |
| Sauce black vs Varadero black | dct | 4037.664766 | 0.689984075 | 0.189669309 | 3.637826694 | 0.000274948 | 0.004406668 |
| Sauce black vs Varadero black | ercc4 | 152.5341623 | -0.915260303 | 0.270058807 | -3.38911481 | 0.000701186 | 0.009248312 |
| Sauce black vs Varadero black | fanca | 454.3875025 | -0.627221197 | 0.186002683 | -3.372108329 | 0.000745951 | 0.009734835 |
| Sauce black vs Varadero black | gart | 509.5060558 | 0.5813854 | 0.183470079 | 3.168829513 | 0.001530541 | 0.017300713 |
| Sauce black vs Varadero black | gfpt1 | 372.7765557 | 0.698466965 | 0.216116416 | 3.231901484 | 0.001229694 | 0.014462878 |
| Sauce black vs Varadero black | gja5 | 361.5906172 | -1.13967172 | 0.210365969 | -5.41756695 | 6.04E-08 | 2.83E-06 |
| Sauce black vs Varadero black | gnas | 186.1328318 | 1.010165364 | 0.266500037 | 3.790488643 | 0.000150351 | 0.00262844 |
| Sauce black vs Varadero black | hdac2 | 635.9642683 | -0.637658509 | 0.212196548 | -3.005037149 | 0.002655484 | 0.026604855 |
| Sauce black vs Varadero black | hps1 | 225.1434415 | 0.665832392 | 0.234623902 | 2.837871097 | 0.004541552 | 0.039947777 |
| Sauce black vs Varadero black | hsp90b1 | 5250.860626 | -0.38190379 | 0.138056099 | -2.766294237 | 0.005669735 | 0.047657346 |
| Sauce black vs Varadero black | ift140 | 188.2680515 | 0.84647519 | 0.278359185 | 3.040945781 | 0.002358363 | 0.024416379 |
| Sauce black vs Varadero black | ift27 | 200.4358597 | -0.85194326 | 0.239947701 | -3.550537293 | 0.000384446 | 0.005780138 |
| Sauce black vs Varadero black | ikbkb | 871.1483097 | 0.515136398 | 0.153736002 | 3.350785698 | 0.000805826 | 0.010311439 |
| Sauce black vs Varadero black | krt1 | 234635.6543 | 1.416934339 | 0.13686698 | 10.35263829 | 4.07E-25 | 2.27E-22 |
| Sauce black vs Varadero black | lrat | 62.37661011 | 1.569768845 | 0.405945606 | 3.866943805 | 0.000110208 | 0.002014945 |
| Sauce black vs Varadero black | mab21l2 | 44.24341606 | -3.028190023 | 0.707957307 | -4.277362477 | 1.89E-05 | 0.000446653 |
| Sauce black vs Varadero black | mcm2 | 1117.736494 | -0.619780944 | 0.167695617 | -3.695868479 | 0.000219136 | 0.003659152 |
| Sauce black vs Varadero black | mlph | 415.8046266 | 1.144382952 | 0.210239635 | 5.443231244 | 5.23E-08 | 2.50E-06 |
| Sauce black vs Varadero black | mmp17 | 94.05660628 | -1.172812353 | 0.339467317 | -3.454860878 | 0.000550577 | 0.007500602 |
| Sauce black vs Varadero black | mpnd | 106.2067942 | -1.44208202 | 0.328586523 | -4.388743667 | 1.14E-05 | 0.000283647 |
| Sauce black vs Varadero black | mpv17 | 186.1242257 | -0.938554021 | 0.276764175 | -3.39116875 | 0.000695952 | 0.009200573 |
| Sauce black vs Varadero black | mpzl3 | 118.186085 | 0.85818834 | 0.305464797 | 2.809450867 | 0.004962609 | 0.042841815 |
| Sauce black vs Varadero black | nmnat1 | 64.59531307 | 1.814502651 | 0.414637426 | 4.376118841 | 1.21E-05 | 0.000297662 |
| Sauce black vs Varadero black | obscn | 11.05606695 | 6.382735535 | 1.822656116 | 3.501886878 | 0.000461976 | 0.006616321 |
| Sauce black vs Varadero black | ovol1 | 254.6136139 | 0.794392847 | 0.227660594 | 3.489373514 | 0.000484154 | 0.006820285 |
| Sauce black vs Varadero black | phactr4-b | 909.0556906 | 0.650386873 | 0.156343155 | 4.159995833 | 3.18E-05 | 0.000716776 |
| Sauce black vs Varadero black | pomgnt1 | 512.8482364 | 0.624864291 | 0.174046727 | 3.59020996 | 0.000330412 | 0.005119319 |
| Sauce black vs Varadero black | prdm1 | 311.4929487 | 1.266590442 | 0.289181195 | 4.379919803 | 1.19E-05 | 0.00029407 |
| Sauce black vs Varadero black | prkci | 324.5975646 | 0.770846276 | 0.248985578 | 3.095947494 | 0.001961851 | 0.021052677 |
| Sauce black vs Varadero black | ptpn6 | 314.4708734 | -1.010429967 | 0.220433072 | -4.583840154 | 4.57E-06 | 0.000127573 |
| Sauce black vs Varadero black | rabggta | 439.0101636 | -1.886835676 | 0.196489837 | -9.60271382 | 7.79E-22 | 3.00E-19 |
| Sauce black vs Varadero black | raph1 | 715.7977352 | -0.686160277 | 0.162269266 | -4.228528871 | 2.35E-05 | 0.000545164 |
| Sauce black vs Varadero black | rbp1 | 327.1672935 | -0.922609896 | 0.275392162 | -3.350167593 | 0.000807627 | 0.010322653 |
| Sauce black vs Varadero black | recql4 | 127.5209307 | -1.166506643 | 0.293037709 | -3.980739019 | 6.87E-05 | 0.001372921 |
| Sauce black vs Varadero black | rho | 39.28872701 | -1.441256544 | 0.479216828 | -3.0075249 | 0.002633845 | 0.026435479 |
| Sauce black vs Varadero black | rp2 | 25.1203528 | 2.433138846 | 0.695627251 | 3.497762404 | 0.000469179 | 0.00668849 |
| Sauce black vs Varadero black | rpe65 | 166.894072 | 0.99367025 | 0.286499827 | 3.468310124 | 0.000523742 | 0.007232049 |
| Sauce black vs Varadero black | sdc4-b | 2096.807067 | 0.508834505 | 0.143151995 | 3.554505169 | 0.000378691 | 0.0057167 |
| Sauce black vs Varadero black | slc12a2 | 935.8476794 | -0.681568503 | 0.185370326 | -3.676794009 | 0.000236184 | 0.003914551 |
| Sauce black vs Varadero black | slc2a1 | 20.17195844 | 4.130512849 | 0.887260002 | 4.65535789 | 3.23E-06 | 9.46E-05 |
| Sauce black vs Varadero black | slc31a1 | 807.4401786 | -0.893142201 | 0.168381692 | -5.304271434 | 1.13E-07 | 4.86E-06 |
| Sauce black vs Varadero black | stxbp1 | 41.12919687 | -1.714135246 | 0.465515551 | -3.682229824 | 0.000231203 | 0.003837692 |
| Sauce black vs Varadero black | sytl2 | 1241.532318 | 0.737613836 | 0.167245704 | 4.410360435 | 1.03E-05 | 0.000261413 |
| Sauce black vs Varadero black | terf2 | 367.1522718 | 1.446723425 | 0.218234326 | 6.629220297 | 3.37E-11 | 3.19E-09 |
| Sauce black vs Varadero black | tgfbr2 | 154.818907 | 1.368622924 | 0.289290749 | 4.73095987 | 2.23E-06 | 6.90E-05 |
| Sauce black vs Varadero black | tshr | 102.7734306 | -3.561501308 | 0.424518742 | -8.389503106 | 4.88E-17 | 1.03E-14 |
| Sauce black vs Varadero black | tspan36 | 486.1981774 | -1.235102954 | 0.25426802 | -4.857484459 | 1.19E-06 | 4.05E-05 |
| Sauce black vs Varadero black | ttc8 | 119.2344053 | 1.191898664 | 0.343482533 | 3.47004156 | 0.000520378 | 0.007203396 |
| Sauce black vs Varadero black | tub | 142.0667149 | -0.785695867 | 0.265754597 | -2.956471407 | 0.003111811 | 0.030070968 |
| Sauce black vs Varadero black | tyr | 2363.236727 | 0.508890993 | 0.155823646 | 3.26581368 | 0.0010915 | 0.013181785 |
| Sauce black vs Varadero black | wnt7a | 352.8220174 | 0.673798577 | 0.214458324 | 3.14186255 | 0.001678768 | 0.018623158 |
| Sauce black vs Varadero black | wrap53 | 652.3854115 | -0.596188984 | 0.202562843 | -2.943229736 | 0.003248073 | 0.030880189 |
| Sauce black vs Varadero black | wrn | 196.527755 | -0.943997335 | 0.235889564 | -4.001861371 | 6.28E-05 | 0.001281965 |
| Sauce black vs Varadero black | ywhaz | 5354.424291 | 0.426256158 | 0.13486111 | 3.160704811 | 0.001573879 | 0.017705744 |
| Sauce black vs Varadero black | zic1 | 195.8949247 | -1.545558522 | 0.37968653 | -4.070617202 | 4.69E-05 | 0.001005363 |
| Sauce black vs Varadero black | zic2-a | 42.18913383 | -1.671786272 | 0.508769152 | -3.285942685 | 0.001016417 | 0.012418266 |
| Sauce black vs Varadero black | cdv3-a | 739.3766646 | 0.47279638 | 0.171283014 | 2.760322624 | 0.005774431 | 0.048283057 |
| Sauce black vs Varadero black | daam1-a | 60.98729904 | 1.765498962 | 0.526655751 | 3.352282698 | 0.000801481 | 0.010267601 |
| Sauce black vs Varadero black | dbnl-a | 71.08109129 | 1.570321775 | 0.445428472 | 3.5254185 | 0.000422814 | 0.006214813 |
| Sauce black vs Varadero black | fcn1-a | 255.8924228 | -1.705070717 | 0.26808896 | -6.360093002 | 2.02E-10 | 1.64E-08 |
| Sauce black vs Varadero black | gamt-a | 102.6874728 | -1.191083524 | 0.375511207 | -3.171898737 | 0.001514458 | 0.017158226 |
| Sauce black vs Varadero black | hsd11b1l-a | 2261.73133 | -0.994335556 | 0.25467931 | -3.90426515 | 9.45E-05 | 0.001792521 |
| Sauce black vs Varadero black | irs2-a | 403.1238298 | 0.994843838 | 0.253184849 | 3.929318216 | 8.52E-05 | 0.001635094 |
| Sauce black vs Varadero black | kdm3a-a | 1890.051962 | -0.499231288 | 0.171718783 | -2.907260817 | 0.003646091 | 0.03358696 |
| Sauce black vs Varadero black | lsm14b-a | 272.453281 | -0.721089379 | 0.222208951 | -3.245095999 | 0.00117411 | 0.013968037 |
| Sauce black vs Varadero black | ncam1-a | 76.66365623 | -1.250506338 | 0.400477175 | -3.122540852 | 0.001792972 | 0.019617329 |
| Sauce black vs Varadero black | scnn1b-a | 434.2786602 | 0.779959228 | 0.247840893 | 3.147015895 | 0.00164946 | 0.018371347 |
| Sauce black vs Varadero black | slc22a6-a | 61.20075418 | 2.219657803 | 0.464331881 | 4.780326085 | 1.75E-06 | 5.55E-05 |
| Sauce black vs Varadero black | tlcd4-a | 622.1618987 | 0.872546032 | 0.195860722 | 4.454931157 | 8.39E-06 | 0.000216506 |
| Sauce black vs Varadero black | zic2-a | 42.18913383 | -1.671786272 | 0.508769152 | -3.285942685 | 0.001016417 | 0.012418266 |
| Sauce black vs Varadero black | gchfr | 46.82704635 | -3.76752074 | 0.596426684 | -6.316821235 | 2.67E-10 | 2.16E-08 |
| Sauce black vs Varadero black | xdh | 1063.619912 | -2.852340665 | 0.486513275 | -5.862821865 | 4.55E-09 | 2.93E-07 |
| Sauce black vs Varadero black | rbp2 | 30.95335526 | 7.055891102 | 1.634850157 | 4.315925267 | 1.59E-05 | 0.000381822 |
| Sauce black vs Varadero black | rbp4 | 29138.73354 | -0.4996193 | 0.173233772 | -2.884075619 | 0.003925645 | 0.035682162 |
| Sauce black vs Varadero black | rdh14 | 1039.406708 | -0.81323979 | 0.220448668 | -3.689021103 | 0.000225119 | 0.003753432 |
| Sauce black vs Varadero black | rxrb | 556.2372063 | 0.76961224 | 0.187419737 | 4.106356416 | 4.02E-05 | 0.000875278 |
| Sauce black vs Varadero black | sult1a1 | 298.0784565 | -2.824443785 | 0.308696085 | -9.149593797 | 5.71E-20 | 1.88E-17 |
| Sauce black vs Varadero black | adcy7 | 150.2393459 | 1.605590096 | 0.308215695 | 5.209306732 | 1.90E-07 | 7.76E-06 |
| Sauce black vs Varadero black | creb3l1 | 1681.862788 | -0.618063986 | 0.169022606 | -3.656694222 | 0.000255489 | 0.004148352 |
| Sauce black vs Varadero black | dvl1 | 123.7581216 | 1.182835519 | 0.354627138 | 3.335434301 | 0.000851663 | 0.010792148 |
| Sauce black vs Varadero black | wnt4 | 145.964154 | 0.866918313 | 0.268915595 | 3.223756185 | 0.001265211 | 0.014753308 |
| All black vs orange/red | adgra2 | 142.8867241 | -1.008688289 | 0.240061362 | -4.201793585 | 2.65E-05 | 0.002895027 |
| All black vs orange/red | arl6ip1 | 1970.623202 | 0.495330674 | 0.119042108 | 4.160970292 | 3.17E-05 | 0.003298321 |
| All black vs orange/red | clcn2 | 46.33134813 | 1.066261299 | 0.289660637 | 3.681070751 | 0.000232257 | 0.015146444 |
| All black vs orange/red | dio2 | 185.8951611 | -0.672989143 | 0.189458456 | -3.552172634 | 0.000382064 | 0.022031023 |
| All black vs orange/red | dmxl2 | 83.30396358 | -0.751802944 | 0.225249272 | -3.337648717 | 0.000844905 | 0.03916804 |
| All black vs orange/red | erbb3 | 96.63656441 | -1.110778502 | 0.290391988 | -3.825100378 | 0.000130719 | 0.010085601 |
| All black vs orange/red | fscn2 | 29.97530673 | 1.420839636 | 0.337117615 | 4.214670404 | 2.50E-05 | 0.002768252 |
| All black vs orange/red | gmps | 730.4056655 | 0.662983196 | 0.128849619 | 5.14540284 | 2.67E-07 | 5.73E-05 |
| All black vs orange/red | hmx1 | 61.96797458 | 1.294891503 | 0.268664675 | 4.81973115 | 1.44E-06 | 0.000246085 |
| All black vs orange/red | impdh1 | 394.8707204 | 1.044603278 | 0.184402159 | 5.664810452 | 1.47E-08 | 4.24E-06 |
| All black vs orange/red | kcnj13 | 54.03420539 | -3.125936331 | 0.380978516 | -8.205019966 | 2.31E-16 | 1.68E-13 |
| All black vs orange/red | mab21l1 | 119.5659475 | 1.217665629 | 0.204214398 | 5.962682572 | 2.48E-09 | 8.50E-07 |
| All black vs orange/red | mab21l2 | 44.24341606 | 2.123791335 | 0.310754286 | 6.834310677 | 8.24E-12 | 3.93E-09 |
| All black vs orange/red | mc1r | 695.0889768 | 1.631196101 | 0.176806257 | 9.22589576 | 2.81E-20 | 2.37E-17 |
| All black vs orange/red | mlana | 1337.680639 | -1.502439159 | 0.130836782 | -11.48330876 | 1.60E-30 | 1.95E-27 |
| All black vs orange/red | mlph | 415.8046266 | 0.736515915 | 0.134740587 | 5.466177138 | 4.60E-08 | 1.17E-05 |
| All black vs orange/red | mreg | 77.65801574 | -1.495493161 | 0.228076701 | -6.556974717 | 5.49E-11 | 2.31E-08 |
| All black vs orange/red | otx1 | 325.5219672 | 1.212231062 | 0.205945771 | 5.886166328 | 3.95E-09 | 1.31E-06 |
| All black vs orange/red | paics | 1979.988922 | 0.385534803 | 0.104508834 | 3.689016416 | 0.000225123 | 0.014858098 |
| All black vs orange/red | pax3-a | 76.10641965 | 2.612349913 | 0.251546151 | 10.38517149 | 2.90E-25 | 3.17E-22 |
| All black vs orange/red | pax7 | 31.79901178 | 4.360887878 | 0.552796619 | 7.888774506 | 3.05E-15 | 1.86E-12 |
| All black vs orange/red | pmel | 2597.184873 | -3.602357688 | 0.111449503 | -32.32277926 | 3.35E-229 | 3.67E-225 |
| All black vs orange/red | ptch1 | 208.2580408 | -0.766780314 | 0.196787868 | -3.896481635 | 9.76E-05 | 0.00848657 |
| All black vs orange/red | rab27a | 347.0421081 | 0.614425395 | 0.134412814 | 4.571181684 | 4.85E-06 | 0.000727871 |
| All black vs orange/red | rbp1 | 327.1672935 | 0.674435255 | 0.170002685 | 3.967203538 | 7.27E-05 | 0.006584546 |
| All black vs orange/red | rlbp1 | 105.1219335 | 4.714741269 | 0.35227892 | 13.38354641 | 7.55E-41 | 1.18E-37 |
| All black vs orange/red | rpl27a | 17775.9955 | 0.483734683 | 0.149975828 | 3.225417656 | 0.00125789 | 0.049573541 |
| All black vs orange/red | rps7 | 18696.52729 | 0.512043908 | 0.153436823 | 3.337164422 | 0.000846379 | 0.03916804 |
| All black vs orange/red | scarb2 | 101.6115492 | -0.819074649 | 0.225075283 | -3.639114165 | 0.000273578 | 0.016918636 |
| All black vs orange/red | slc16a2 | 31.45020333 | -1.800999135 | 0.455696775 | -3.952187584 | 7.74E-05 | 0.006897826 |
| All black vs orange/red | slc22a7 | 284.8400126 | 5.662294871 | 0.276513506 | 20.47746223 | 3.42E-93 | 1.25E-89 |
| All black vs orange/red | slc24a5 | 153.4310886 | -1.025987145 | 0.162035569 | -6.331863765 | 2.42E-10 | 9.48E-08 |
| All black vs orange/red | slc45a2 | 76.60674496 | -1.336977486 | 0.257831056 | -5.185478845 | 2.15E-07 | 4.82E-05 |
| All black vs orange/red | sox18 | 111.3756057 | -0.817061181 | 0.243962552 | -3.349125402 | 0.000810671 | 0.038448965 |
| All black vs orange/red | tfec | 8.800242614 | 2.848995241 | 0.811106554 | 3.51247962 | 0.000443946 | 0.024407293 |
| All black vs orange/red | trpm1 | 221.0996035 | 0.88265614 | 0.159155484 | 5.545873247 | 2.92E-08 | 7.82E-06 |
| All black vs orange/red | trpm7 | 125.8493994 | -0.827876215 | 0.206060481 | -4.017637012 | 5.88E-05 | 0.005589813 |
| All black vs orange/red | tyrp1 | 585.6352333 | -4.396123411 | 0.191282779 | -22.98232719 | 7.00E-117 | 3.84E-113 |
| All black vs orange/red | usp43 | 156.1667479 | -0.734989225 | 0.198063369 | -3.710879137 | 0.000206541 | 0.014142875 |
| All black vs orange/red | dapl1-a | 1590.020249 | 0.665707863 | 0.172817683 | 3.852081866 | 0.000117118 | 0.009504764 |
| All black vs orange/red | pax3-a | 76.10641965 | 2.612349913 | 0.251546151 | 10.38517149 | 2.90E-25 | 3.17E-22 |
| All black vs orange/red | tmem159-a | 201.028125 | 0.551327246 | 0.168257029 | 3.27669666 | 0.001050291 | 0.044139991 |
| All black vs orange/red | gch1 | 3744.25712 | 5.523316767 | 0.359693278 | 15.35562966 | 3.25E-53 | 5.93E-50 |
| All black vs orange/red | xdh | 1063.619912 | 4.610348567 | 0.272180312 | 16.93858214 | 2.34E-64 | 6.40E-61 |
| All black vs orange/red | cyp26b1 | 109.0335882 | -0.88349096 | 0.270360222 | -3.267828946 | 0.001083758 | 0.044139991 |
| All black vs orange/red | dgat2 | 128.8793382 | 0.655641208 | 0.191761551 | 3.419044145 | 0.000628415 | 0.031153475 |

**Table S3:** Number of *Ranitomeya imitator* froglets used from each established color morph breeding pair.

| **Color morph breeding pairs** | **Skin patches** | **Number of froglets** |
| --- | --- | --- |
| Sauce pair 1 | Black & orange | 5 |
| Sauce pair 2 | Black & orange | 5 |
| Sauce pair 3 | Black & orange | 5 |
| Sauce pair 4 | Black & orange | 1 |
| Varadero pair 1 | Black & red | 5 |
| Varadero pair 2 | Black & red | 5 |
| Varadero pair 3 | Black & red | 5 |
| Varadero pair 4 | Black & red | 1 |

**Supplementary Figures**

**
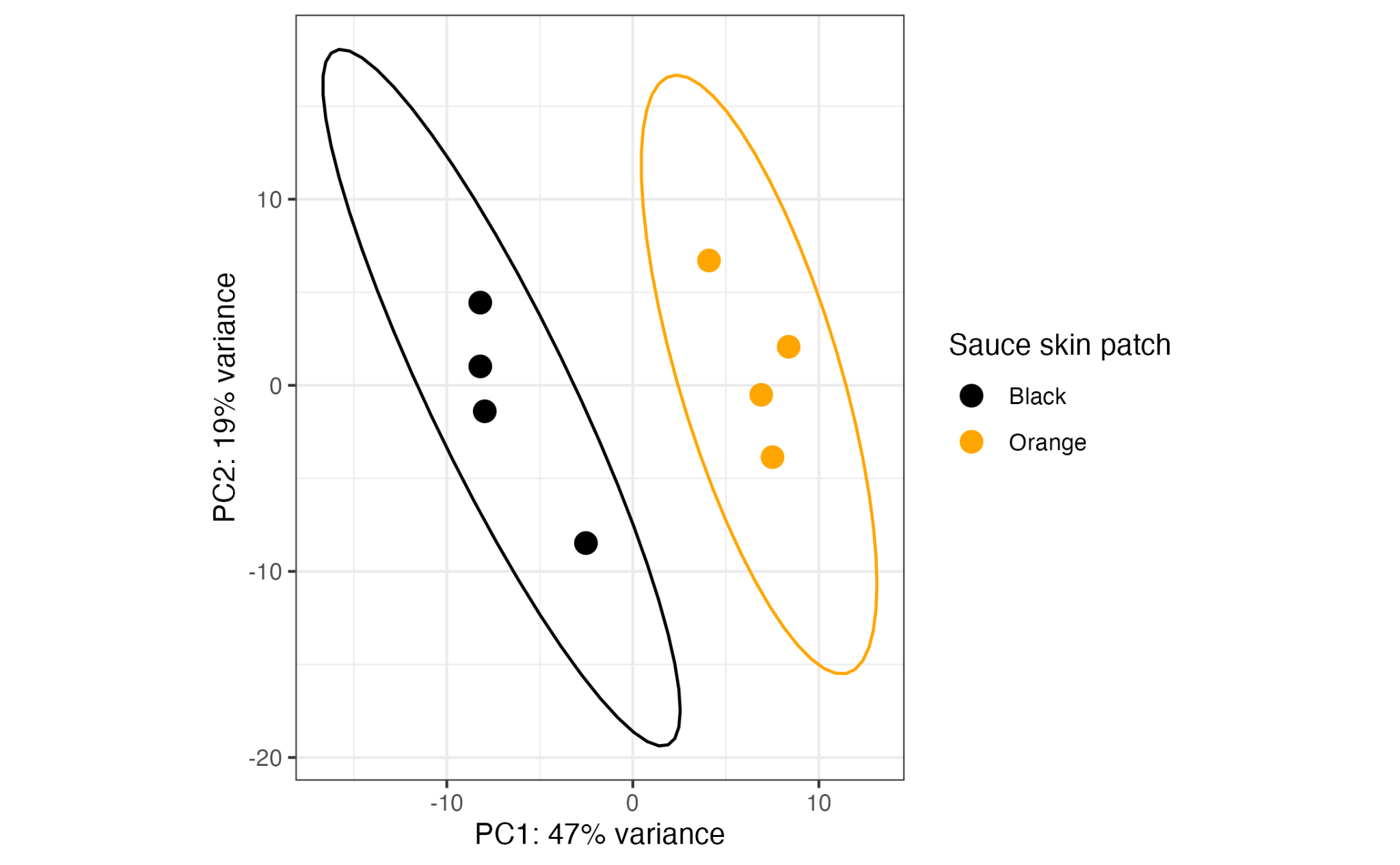
**

**Figure S1:** Plot of the principal component analysis summarizing the expression patterns across samples of *Ranitomeya imitator:* Black and orange skin color patches from the Sauce morph. The ellipses are derived by a multivariate t-distribution and group skin color patches.


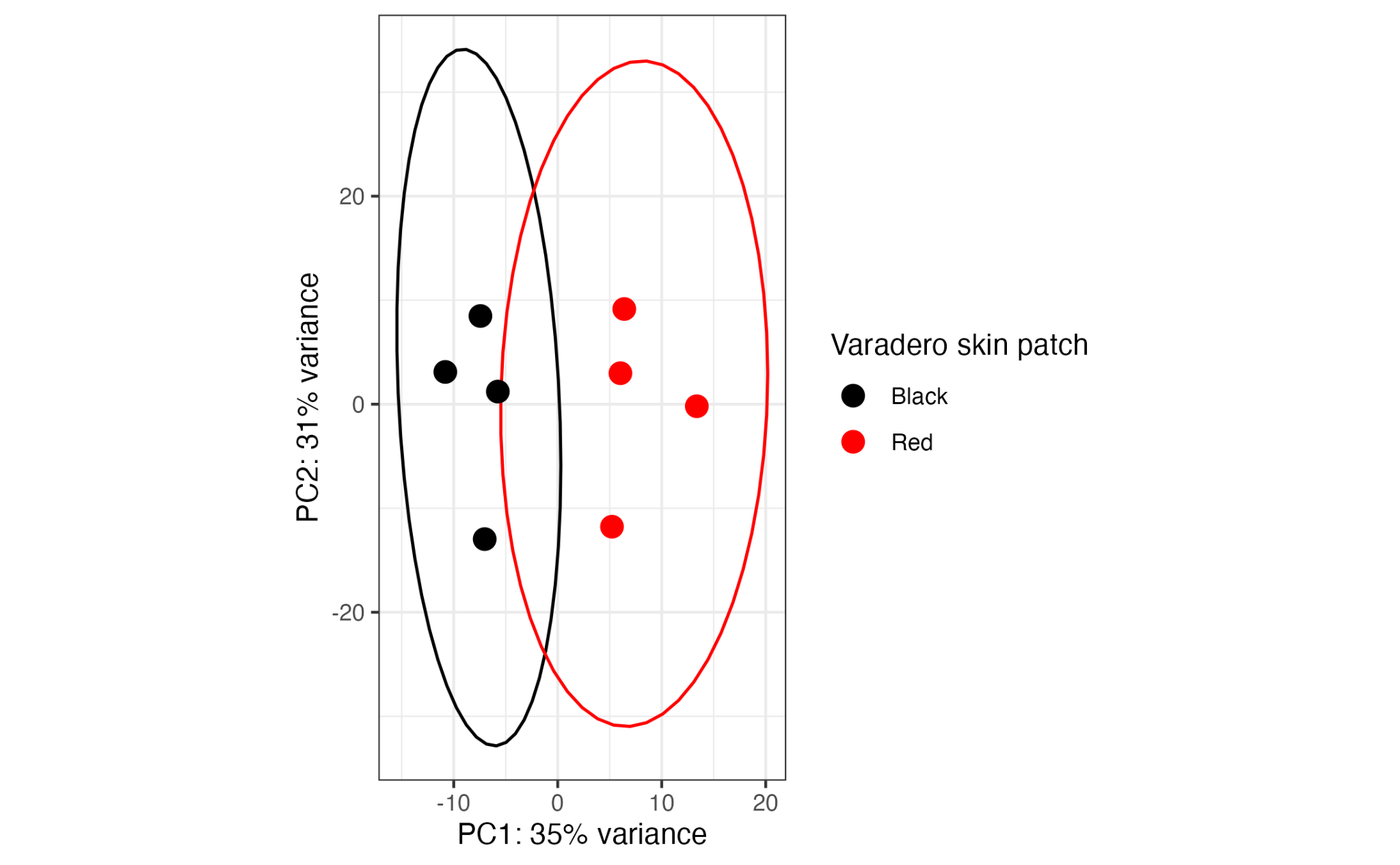


**Figure S2:** Plot of the principal component analysis summarizing the expression patterns across samples of *Ranitomeya imitator:* Black and red skin color patches from the Varadero morph. The ellipses are derived by a multivariate t-distribution and group skin color patches.


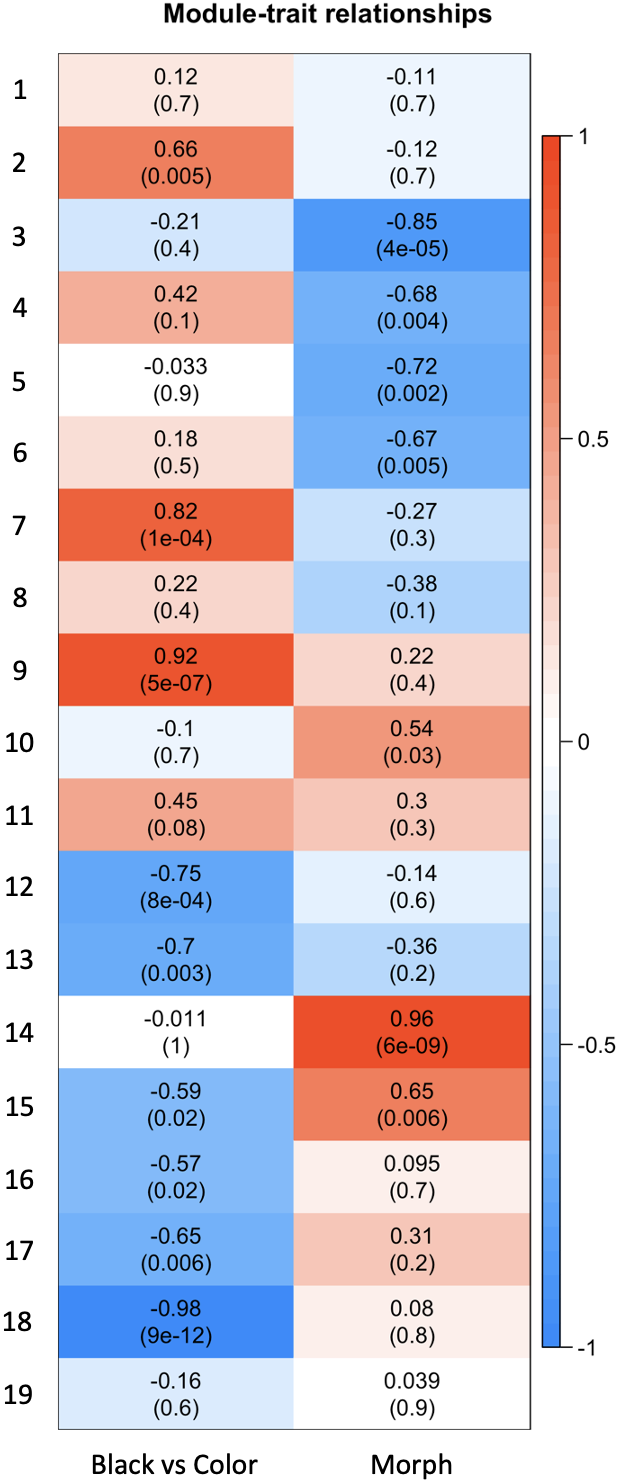


**Figure S3** Weighted gene co-expression network analysis (WGCNA) of RNAseq data from all black and color (orange and red) skin patches in *Ranitomeya imitator*. The R value is represented on the top, whereas the adjusted p-value is represented on the bottom.
